# MnM-W-MMGBSA: A Computational Strategy to Improve Relative Binding Free Energies of Protein–Protein Interaction Systems

**DOI:** 10.1021/acs.jpcb.5c04085

**Published:** 2025-11-30

**Authors:** Md Nazmul Hasan, Shilpa Sharma, Justice Josiah Mallen, Arjun Saha

**Affiliations:** Department of Chemistry and Biochemistry, 118733 University of Wisconsin-Milwaukee, Milwaukee, Wisconsin 53211, United States

## Abstract

Protein–protein interactions (PPIs) have become increasingly attractive as therapeutic targets due to their central role in regulating cellular functions. Despite computational advancements, accurately estimating binding free energies for PPIs remains challenging due to the dynamic and critically solvent-exposed nature of their interfaces. In this study, we present MnM-W-MMGBSA (i.e., MM-GBSA with Mix-and-Match sampling and water inclusion), a method that addresses these challenges by incorporating both conformational flexibility and interfacial solvation effects. We thoroughly demonstrated the applicability of MnM-W-MMGBSA across a diverse set of 20 PPI systems and validated its robustness using two distinct and rigorous simulation schemes. We demonstrate that our protocol improves correlation with experimental binding affinities—from 47% to 70% with MnM sampling alone for standard MM-GBSA, and up to 89% when interfacial water molecules are included. Our approach underscores the pivotal role of individual protein dynamics in validating the concept of “destabilization of individual proteins in the unbound form.” Specifically, we show that in explicit solvent, such destabilization leads to a loss of native structure, suggesting that excessive conformational sampling may compromise the accuracy of binding affinity predictions. Furthermore, the critical role of intrinsically disordered regions in the interface of PPIs, as well as the impact of the MnM approach in the pairwise per-residue energy decomposition, were also investigated. Finally, our implementation overcomes the limitations of the gmx_MMPBSA tool for incorporating explicit solvent molecules from MD simulation trajectories into the complex/receptor during MM-GBSA calculation, providing an automated and reproducible workflow using GROMACS with AmberTools to enable efficient high-throughput screening of protein–protein complexes. The protocol is robust, computationally efficient, and applicable to a broad range of PPIs. Overall, our protocol offers a practical and physically meaningful alternative for estimating the binding affinities of PPIs and provides a valuable tool for advancing peptide-based drug discovery.

## Graphical abstract

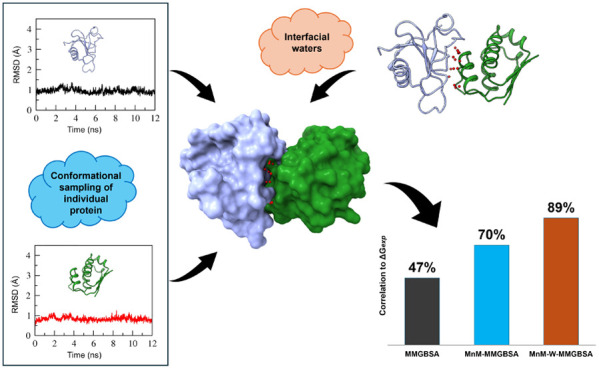

## 1 Introduction

Protein–protein interactions (PPIs) are fundamental to virtually every biological process, including enzymatic regulation, immune surveillance, cell cycle control, signal transduction, and gene expression regulation.^1–3^ The human interactome is estimated to encompass over 650 000 unique PPIs, representing an expansive and largely underexplored landscape for therapeutic intervention.^4^ Despite this immense potential, targeting PPIs remains an immense challenge due to the structural characteristics of their interfaces, which are typically large, shallow, and predominantly hydrophobic.^5,6^ These features contrast sharply with the well-defined binding pockets observed in traditional drug targets, such as enzymes or G protein-coupled receptors, complicating both experimental and computational investigations of PPIs.^7,8^

Computational estimation of binding free energies is an essential step in characterizing molecular interactions and predicting the stability of biomolecular complexes.^9^ However, for PPIs, accurate free energy prediction is particularly difficult due to the dynamic nature of protein interfaces and the absence of deep binding clefts.^10,11^ One of the central challenges is the substantial conformational flexibility observed in PPI complexes.^12^ Unlike small molecule–protein interactions, PPI binding often induces significant rearrangements in both backbone and side-chain orientations, which can dynamically modulate the shape and surface of the interface.^12^ Additionally, the behavior of interfacial water molecules—which can mediate crucial hydrogen bonds—adds another layer of complexity.^10,13^ Ignoring such conformational and solvent dynamics may result in energetically incomplete or misleading predictions.

Multiple studies have demonstrated that structural rearrangements and induced fit mechanisms can significantly impact the accuracy of computed binding free energies for protein–protein systems.^14–16^ Hence, the comprehensive sampling of conformational ensembles is critical to capture the energetic landscape of PPI binding events. Molecular dynamics (MD) simulations offer a powerful approach to address this issue, allowing the capture of time-dependent conformational sampling that is inaccessible to static structural models.^17,18^ Importantly, accurate estimation of binding energetics also requires sampling the conformational space of the individual unbound protein chains.^19–21^ Neglecting this component can result in incomplete modeling of the entropic contributions and conformational penalties associated with complex formation.^22,23^ Studies have shown that including both bound and unbound conformational ensembles significantly improves the reliability of binding free energy predictions for PPIs.^24–26^ Thus, capturing these dynamic rearrangements is essential for a realistic representation of binding thermodynamics in PPI systems. Absolute binding free energy (ABFE) estimation using more theoretically rigorous alchemical or geometric methods incorporates conformational sampling and enables an accurate prediction of binding free energies. For example, Fu et al. accurately determined the absolute binding free energy (ABFE) for both protein–ligand and protein–peptide systems using a theoretically rigorous approach.^27^ While this method offers high accuracy, it is computationally expensive and time-consuming, making it impractical for relative binding free energy (RBFE) calculations during large-scale virtual screening, particularly in complex systems such as protein–protein interactions (PPIs).

Among various free energy estimation methods, the Molecular Mechanics Generalized Born Surface Area (MM-GBSA)^28,29^ approach is one of the most popular due to its balance of computational efficiency and reasonable accuracy.^30–35^ MM-GBSA has been shown to perform well in systems involving small-molecule ligands or short peptides.^31,33,36–44^ However, its applicability is largely restricted to closely related congeneric ligand series, and it faces major limitations in prospective lead optimization studies because it neglects critical factors such as explicit solvent effects, entropic contributions, and ligand strain.^45^ For instance, Coveney et al. demonstrated that MM-GBSA/PBSA exhibits substantial limitations in ranking clinically relevant HIV-1 protease inhibitors.^46^ Similarly, Mobley et al. reported its poor performance in reproducing experimental binding free energies across multiple D3R Grand Challenges.^47^ Moreover, when applied to PPIs, it often yields poor correlation with experimental binding affinities.^48^ This is largely attributed to the increased dimensionality of the conformational space and the critical role of solvent, particularly interfacial water molecules mediating the interactions.^49,50^ Explicit water molecules, especially those forming bridging hydrogen bonds at the interface, can have significant energetic contributions to PPI stability.^13,51^ One of the ways to model the solvent and bridging hydrogen bonding effect is to retain crystallographic waters during MD simulation for MM-GBSA calculations; however, in the course of MD simulation, these bridging waters are often replaced by nearby solvent molecules.^52^ Several studies have proposed that incorporating dynamically selected interfacial water molecules, especially those consistently residing near key interaction hotspots, can markedly enhance the predictive power of MM-GBSA.^53–55^ For example, Contini et al. improved the consistency and correlation of MM-GBSA-derived binding energies with experimental data by including a fixed number of solvent molecules in the receptor,^56–58^ but the proper conformational sampling of individual binding partners remained a challenge.

In this study, we primarily focused on resolving the sampling limitations of MM-GBSA by implementing a modified protocol, termed MnM-W-MMGBSA (e.g., Mix-and-Match-Water-MMGBSA). This approach has been tested on a data set of 20 PPI complexes encompassing both fully resolved structures and those with partially missing residues (Table 1). The key innovation of our approach lies in the development of the **Mix-and-Match (MnM)** method, which explicitly accounts for the conformational flexibility of the individual binding partners. In MnM, protein–protein interactions are reconstructed by integrating the most representative conformers from molecular dynamics (MD)–derived ensembles of each unbound partner in explicit solvent using population-weighted clustering to capture the conformational variability. This provides a less expensive way of exploring the conformational flexibilities of the PPI landscape with better correlation to the experimental binding affinities compared to the regular MM-GBSA approach. In the present study, we first investigated how incorporating MnM-derived conformational sampling improves the correlation between MM-GBSA-predicted binding free energies and experimental data relative to simulations performed directly from the crystal structures. This offers an alternative strategy to address the conformational challenges inherent in PPI systems. On top of that, we have implemented the inclusion of a fixed number of interfacial water molecules as proposed by Contini and Maffucci.^56^ Contini and Maffucci demonstrated that the inclusion of 30 explicit water molecules from the MD simulation that are closest to the interfacial residues of the PPI complex significantly improves (approximately by 30%) the correlation to experimental Δ*G.* ^56^ Inspired by that study, we also implemented the same strategy in our MnM complexes and included 30 interfacial waters as a part of the receptor to improve the correlation of MM-GBSA-predicted values with the experimental Δ*G* of the PPI systems. We systematically compared the correlation (*r* ^2^) between MM-GBSA-predicted and experimental binding free energies for simulations initiated from crystal structures versus MnM-derived ensembles, both with and without interfacial water molecules, to assess the impact of conformational sampling and solvent representation on prediction accuracy. Recently, we demonstrated the applicability of the MnM method in exploring the binding free energy landscape of a PPI system involving intrinsically disordered regions, specifically for the AF9–BCOR complex implicated in leukemia.^59^

**Table 1:** Selected PPI Complexes with X-ray Crystal Resolutions and Their Experimental Binding Affinities^a^

PDB ID	Resolution (Å)	exp. Δ*G*_bind_ (kcal/mol)
1ACB^60^	2.0	–13.1
1ZHI^61^	2.7	–9.1
1AVX^62^	1.9	–12.5
2HLE^63^	2.05	–10.1
1AY7 ^64^	1.7	–13.2
2HRK^65^	2.05	–11.0
1BVN^66^	2.5	–15.1
2OOB^67^	1.9	–5.7
1EMV^68^	1.7	–18.6
2OUL^69^	2.2	–12.0
1FLE^70^	1.9	–12.3
2SIC^71^	1.8	–13.8
1GLA^72^	2.6	–6.8
2SNI^73^	2.1	–16.0
1KAC^74^	2.6	–10.7
2UUY^75^	1.15	–11.3
1R0R^76^	1.1	–14.2
3BZD^77^	2.3	–9.6
1YVB^78^	2.7	–11.2
3SGB^79^	1.8	–14.5

a Δ*G*_bind_ values were obtained from experimental *K*_D_/*K*_i_/IC_50_ values by using eq 7 (*vide infra*).

## 2 Methods

### 2.1 Structure Preparation

To test the applicability of MnM protocol, a diverse data set of 20 PPIs was selected (Table 1). The X-ray crystal structures of the selected complexes were obtained from the RCSB PDB, with the resolution of structures ranging from 1.1 to 2.7 Å. Structures were processed by removing all crystallographic water molecules and any nonstandard residues or ligands. Additionally, any missing residues in the PPIs were predicted with AlphaFold^80^ for PDB IDs: 1ACB, 1GLA, and 1ZHI. To facilitate analysis, the protein chain with the higher residue count was designated as chain A (residues renumbered starting from 1), while the other was assigned as chain B (residues renumbered sequentially after the last residue of chain A). Figure 1 showcases visual insights into the structural diversity of the studied PPI complexes with relevant color coding of chains. Structure preparation was carried out using the “Structure Preparation” and “Protonate3D” tools in MOE.^81^ Proteins with more than three missing residues at either end were capped with acetyl (N-terminus) and methylamino (C-terminus) groups. Protonation was performed under physiological conditions (pH = 7.4, *T* = 300 K, and salinity = 0.1 M), and a brief energy minimization was applied to resolve steric clashes within the protein complex.

**Figure 1:**
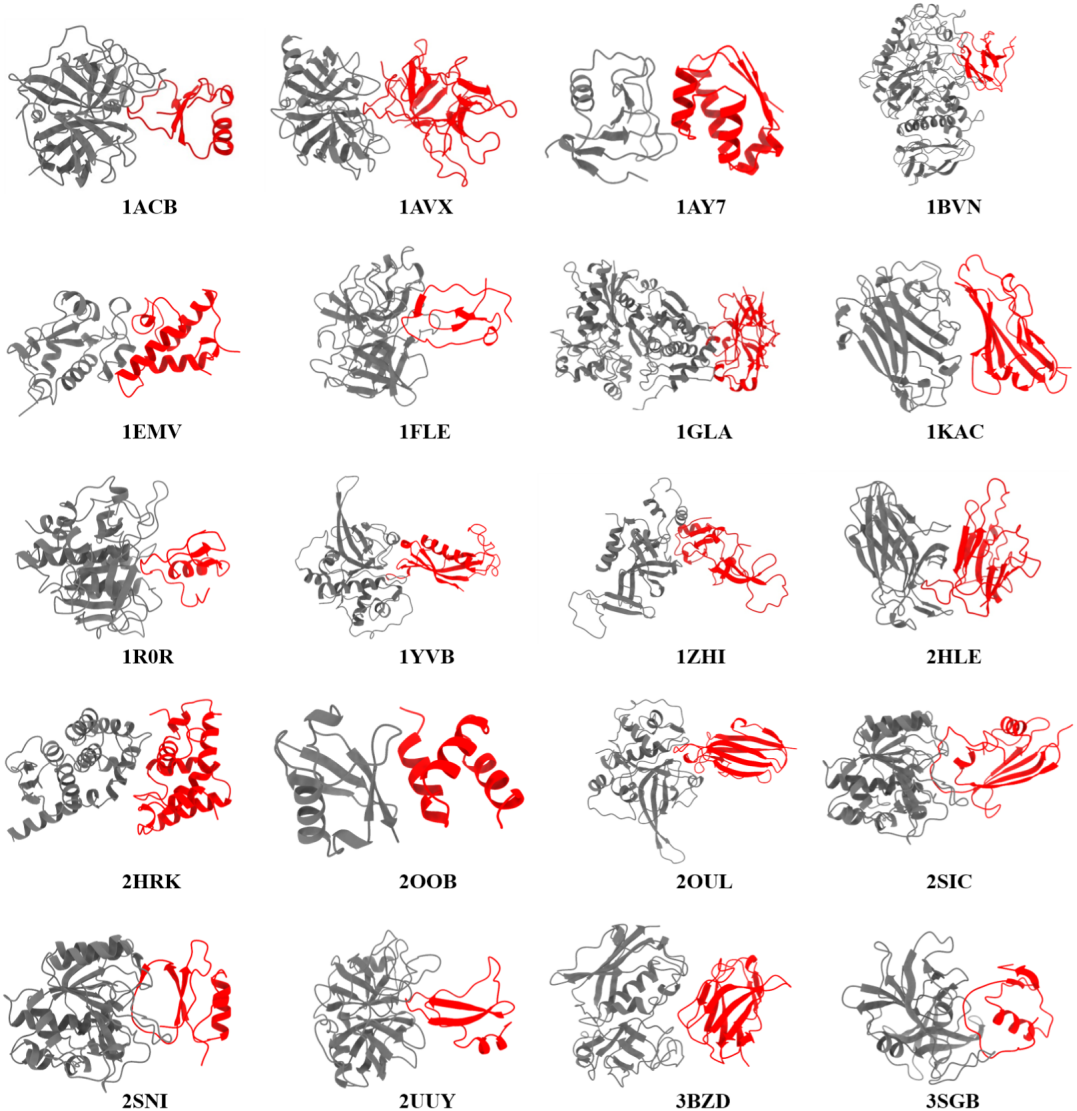
PPI complexes used to evaluate the MnM-W-MMGBSA protocol. For each PPI complex, chain A is represented in gray color, and chain B is represented in red color.

### 2.2 Molecular Dynamics Simulations for Crystal Structures

Structures prepared through the MOE were used as the starting geometries for all-atom molecular dynamics (MD) simulations. GROMACS 2023 package^82^ was employed for running simulations with the ff14SB^83^ force field. This force field has been shown to perform well in simulations of PPI systems.^84,85^ Each complex was solvated in a cubic box of TIP3P water molecules, extending 10 Å beyond any protein atom, and was neutralized with an appropriate number of Na^+^/Cl^–^ ions. The Particle Mesh Ewald (PME) method was used for long-range electrostatics with a cutoff of 12 Å.^86^ Similarly, the van der Waals cutoff was set to 12 Å. Throughout the simulations, periodic boundary conditions (PBCs) were applied. Systems were initially energy minimized through the steepest descent algorithm for 50 000 steps to eliminate unfavorable contacts. Then two-step equilibrations were performed: first, a 1 ns constant volume and temperature (NVT) ensemble for temperature stabilization, followed by a 1 ns constant pressure and temperature (NPT) ensemble for pressure stabilization. The leapfrog algorithm was used with a time step of 2 fs to advance the trajectories. Finally, a 4 ns production run was conducted under the NPT ensemble, with a V-rescale thermostat^87^ and Parrinello–Rahman barostat^88^ for temperature and pressure control, respectively. Bond lengths and angles were constrained by using the LINCS algorithm, and simulation trajectories were recorded every 10 ps. Root Mean Square Deviation (RMSD) from the starting coordinates was used to check the convergence of the MD simulation. This simulation protocol will be referred to as Scheme-0.

### 2.3 Generating Representative Structures for Mix-and-Match (MnM) and Subsequent MD Simulations

One of the prime goals of MnM was to efficiently perform the conformational sampling of the individual binding partners of the PPI complexes. For this purpose, individual protein chains (chains A and B) were separately simulated for a total of 100 ns. The same protocol outlined in the Section 2.2 was followed here. The GROMOS clustering described by Daura et al.^89^ is one of the most effective algorithms for evaluating structural similarities in MD trajectories based on RMSD. A Cα RMSD cutoff value of 0.1 nm was used to determine the structurally similar clusters in the trajectory of each individual protein chain. A schematic representation of the MnM-W-MMGBSA process is shown in Figure 2. As illustrated in Figure 2, two different time intervals from the simulation were selected for clustering. In the first scheme (MnM-Scheme-1), clustering was performed over the first 12 ns of the trajectory, while in MnM-Scheme-2, clustering was done on the last 20 ns (80 to 100 ns). Then, the most populated structures for each chain were extracted from the trajectory for each scheme (Table 2). These frames were then aligned with the backbone of their corresponding crystal structures using UCSF ChimeraX^90^ to generate the Mix-and-Match (MnM) complexes. As these newly generated complexes might have clashes or improper geometry, the “Structure Preparation” and “Protonate3D” tools in MOE^81^ were used to prepare the starting geometry for MD simulations. Finally, the MnM complexes were simulated for 50 ns, following the same protocol as in the Section 2.2.

**Figure 2:**
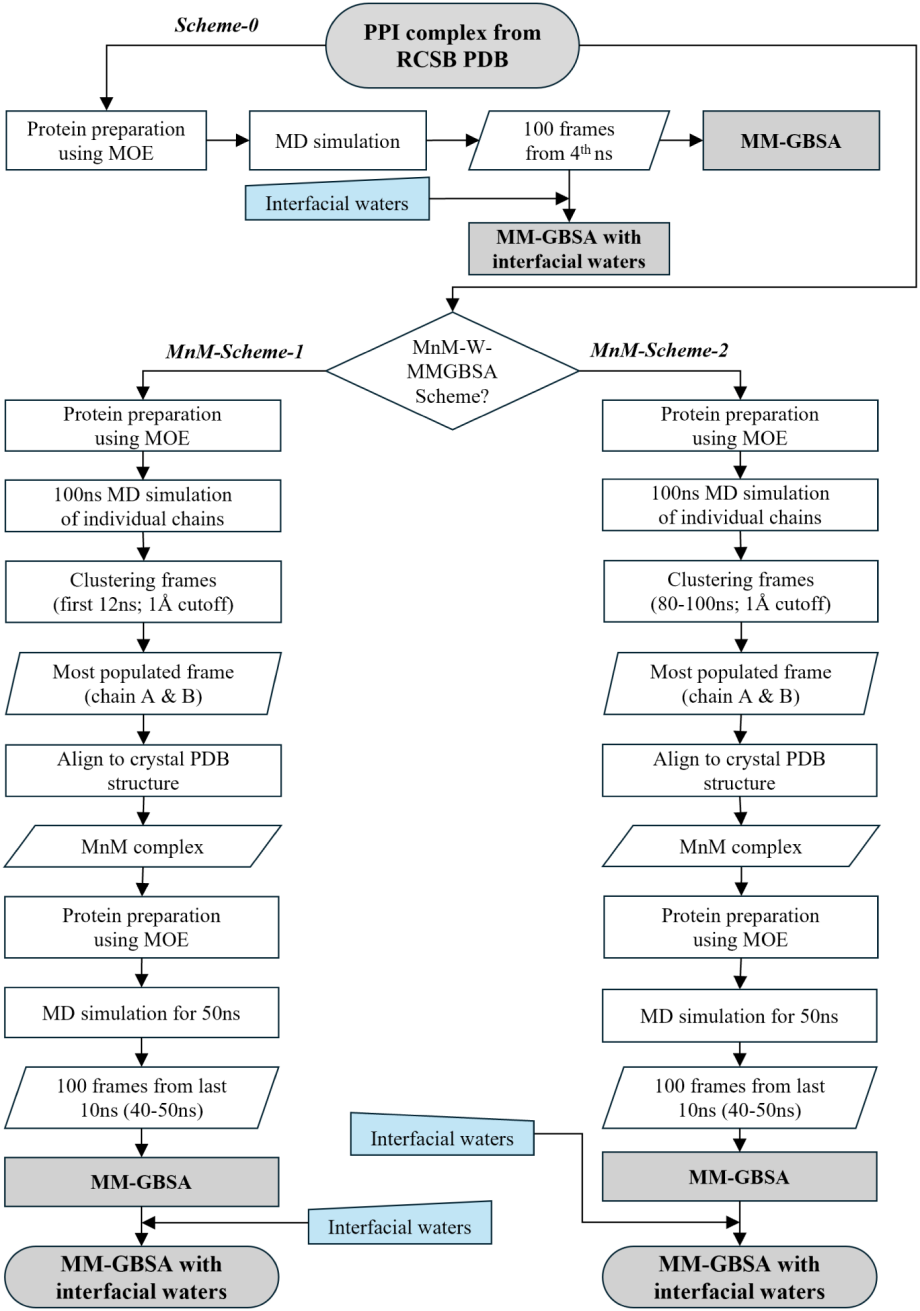
Schematic representation of the schemes for the MnM-W-MMGBSA protocol.

**Table 2:** Most Representative Frame for Each Chain of Each PPI Complex Based on *Gromos* Clustering with C-Alpha RMSD Cutoff of 1 Å^a^

	Chain A representative frame (ps)		Chain B representative frame (ps)	
PDB ID	MnM-Scheme-1	MnM-Scheme-2	MnM-Scheme-1	MnM-Scheme-2
1ACB	9430	91 750	5130	87 870
1AVX	5040	92 340	10 220	94 360
1AY7	7680	89 160	5410	98 750
1BVN	8240	86 710	6520	95 770
1EMV	5790	92 680	10 210	98 620
1FLE	9140	95 340	9780	87 130
1GLA	10 180	91 150	4760	84 690
1KAC	6310	83 070	6580	94 940
1R0R	7780	97 140	3750	80 300
1YVB	5480	87 470	8270	97 240
1ZHI	8870	83 210	9220	87 310
2HLE	8240	90 840	5820	90 510
2HRK	1680	92 530	5290	84 350
2OOB	4190	81 580	3000	91 930
2OUL	7270	85 800	9320	88 520
2SIC	3080	89 920	2170	84 480
2SNI	5460	84 450	4680	91 540
2UUY	6920	88 000	8930	87 330
3BZD	10 780	96 010	8310	88 900
3SGB	8990	90 490	9160	98 470

a Numbers in each scheme represent the corresponding picosecond (ps) of the simulation time frame.

### 2.4 Calculation of Structural Properties

Comprehensive analysis of the MD trajectories was carried out via various GROMACS tools to evaluate the properties of the PPI complexes. Convergence and stability of the trajectories were evaluated by calculating the RMSD of the Cα atoms. Prior to calculating RMSD, frames were aligned to the Cα atoms of the initial coordinates. Solvent-accessible surface area (SASA) was calculated through PyMOL.^91^ Water occupancy in the interface was analyzed using UCSF Chimera.^92^ All the GROMACS topologies were converted to Amber topologies using *parmed* from the AmberTools23 ^93^ package. Other tools such as Visual Molecular Dynamics (VMD),^94^ XMGRACE,^95^ UCSF ChimeraX,^90^ and PyMOL^91^ were utilized to create detailed visual representations and aid in the interpretation of molecular interactions. Interfacial residues were identified robustly and reproducibly using the “InterfaceResidues” PyMOL script.^96^

### 2.5 Performing MM-GBSA Calculations

The MMPBSA.py script from the AmberTools23 ^93^ package was used to perform MM-GBSA analysis for all the structures. The single trajectory-based end-point free energy method of MM-GBSA was followed for the calculation of the binding free energy. The thermodynamic cycle illustrated in Figure S1 was followed to calculate the binding free energy. The binding free energy of a complex is given as:

1 $$\Delta G_{\mathrm{bind}}=G_{\mathrm{complex}}-G_{\mathrm{receptor}}-G_{\mathrm{ligand}}$$

It can be decomposed into contributions from different interactions and expressed as:

2 $$\Delta G_{\mathrm{bind}}=\Delta H-T\Delta S$$

3 $$\Delta G_{\mathrm{bind}}=\Delta E_{\mathrm{MM}}+\Delta G_{\mathrm{solvation}}-T\Delta S$$

The molecular mechanics energy term (Δ*E*_MM_) was calculated using ff14SB^83^ force field, which is a combination of bonded (bonds, angles, dihedrals) and nonbonded (van der Waals and electrostatic) energy terms in the gas phase:

4 $${\Delta E}_{\mathrm{MM}}=\left({\Delta E}_{\mathrm{bonds}}+{\Delta E}_{\mathrm{angle}}+{\Delta E}_{\mathrm{dihedral}}\right)+{\Delta E}_{\mathrm{vdW}}+{\Delta E}_{\mathrm{electrostatic}}$$

Here, “gas phase” refers to the calculation performed on configurations extracted from the molecular dynamics trajectories that were simulated in explicit water after the solvent and counterions were stripped off.

The Δ*G*_solvation_ is composed of both polar and nonpolar components of the solvation free energy.

5 $$\Delta G_{\mathrm{solvation}}=\Delta G_{\mathrm{polar}}+\Delta G_{\mathrm{nonpolar}}$$

The polar component of the solvation free energy (Δ*G*_polar_) was estimated using the GB-Neck2 ^97^ (igb = 8) implicit solvent model, and the nonpolar component (Δ*G*_nonpolar_) was calculated using the following equation:

6 $$\Delta G_{\mathrm{SA}}=\gamma \times \Delta \mathrm{SASA}+b$$

Here, the change in the solvent-accessible surface area upon complex formation was denoted by ΔSASA, with empirical constants γ = 0.0072 kcal·Å^–2^·mol^–1^ and *b* = 0, as defined for the GB-Neck2 model.

The mbondi3 ^97^ radii set and a 0.15 M salt concentration were used for GB calculations. These parameters showed optimum performance in previous studies on evaluating PPI binding free energies by Contini and Maffucci.^56^ For MnM-W-MMGBSA, explicit water molecules should be extracted from the MD simulation trajectory and included in the topology of the complex, as well as the receptor. Currently, the *gmx_MMPBSA* script implemented in GROMACS does not support explicit water molecules in receptor/complex structures for MM-GBSA calculations. For this purpose, the GROMACS topologies and simulated trajectories were converted to Amber *prmtop* and *mdcrd* formats using the *parmed* and *cpptraj* tools from AmberTools23.^93^ Eventually, for all of the schemes, 100 evenly spaced frames were extracted to calculate MM-GBSA. For the simulations starting from the crystal structures (Scheme-0), 100 frames were taken from the 4th ns to calculate MM-GBSA. In MnM-Scheme-1 and MnM-Scheme-2, 100 frames from the last 10 ns (40–50 ns) of the simulation were taken for MM-GBSA calculation. In all the schemes, MM-GBSA was calculated both without and with the inclusion of 30 explicit water molecules from the MD simulations. These waters were selected using cpptraj’s^98^ “closest” command, which retains the 30 solvent molecules nearest to the center of mass of the interfacial residues in the PPI complex. The experimental *K*_D_/*K*_i_/IC_50_ values were converted into binding free energies employing eq 7. This transformation enables a thermodynamic interpretation of binding free energy, allowing for direct comparison between computationally predicted and experimentally determined binding free energy.

7 $$\Delta G\approx RT\ln\left(K_{D}\right)$$

Here, Δ*G* represents the Gibbs free energy of binding; *R* is the universal gas constant; *T* is the absolute temperature in Kelvin, and *K*_D_ denotes the dissociation constant. In the PPIs for which *K*_D_ was not available, other affinity (*K*_i_/IC_50_) values were used.

Previous studies reported that including explicit water as part of the ligand (smaller protein) increases standard deviations without significantly improving correlation with experimental binding free energies.^52^ Therefore, the water molecules closest to the interface were included as part of the receptor. Residues were defined as interfacial if their solvent-accessible surface area (SASA) difference between the complex and the isolated chain exceeded 0.50 Å^2^ (Table S1). After identifying the interfacial residues using the “InterfaceResidues” PyMOL script, “*cpptraj*” calculates the center of mass (COM) of these residues and determines which explicit water molecules from the MD simulation are closest to this COM. It then retains the user-specified number of water molecules (e.g., 30 waters) and removes all other water molecules before performing the MM-GBSA calculation. In a previous study, Contini et al. explored optimal strategies for defining interfacial residues to capture the “closest” waters. They found no significant difference in correlation, whether the retained waters were closest to polar residues or to all interfacial residues. Therefore, in this study, we included 30 explicit waters from the MD simulations that were “closest” to all interfacial residues. The correlation between experimental binding free energy and calculated MM-GBSA (*r* ^2^) was used as the evaluation parameter.

## 3 Results and Discussion

In this study, we implemented the MnM-W-MMGBSA protocol to improve the estimation of binding free energies in protein–protein interaction systems. This approach integrates mix-and-match (MnM)-based conformational sampling with the inclusion of interfacial water molecules retained throughout the trajectory. The rationale behind this method stems from the known limitations of traditional MM-GBSA workflows, which often neglect the contribution of transient water-mediated interactions and conformational variability at the binding interface. By systematically sampling the most populated conformations and retaining the nearest interfacial water molecules on a per-frame basis, MnM-W-MMGBSA aims to capture both structural and solvent-driven components of molecular recognition. Throughout our analyses, we focused on the Generalized Born (GB) model for solvation, which proved to be both robust (less sensitive to simulation details)^17^ and computationally efficient compared to the Poisson–Boltzmann (PB) model. The following sections detail our observations and analyses across multiple systems and highlight the performance and advantages of the MnM-W-MMGBSA protocol.

### 3.1 Effect of Conformational Sampling

X-ray crystallographic structures are often considered to reside in energetically favorable configurations near the global minimum.^99,100^ But for PPIs, the interaction surface is typically larger than conventional protein–ligand systems, and significant structural rearrangements are often observed upon binding.^12^ To explore the conformational flexibility of individual protein partners, we performed the MnM strategy to efficiently sample binding conformations of PPI complexes. The first goal here was to improve the RBFE for PPI systems using a standard MM-GBSA protocol without the inclusion of interfacial water molecules. Binding free energies were calculated using the standard MM-GBSA protocol at the 4th ns of trajectories initiated from the crystal conformations (Scheme-0). This time frame was selected based on the Cα RMSD, which showed convergence at 4 ns for these trajectories (Figure S2). The predicted MM-GBSA values exhibited poor correlation with experimental binding free energies (*r* ^2^ = 0.47; Figure S3A, Table S2). We investigated whether starting structures generated by MnM-Scheme-1 could improve this correlation. The Cα RMSDs for individual protein chains considered for Scheme 1 are shown in Figure 3. Initially, MM-GBSA was calculated in the 4th ns of MnM-Scheme-1 simulations. There was no significant improvement in the correlation to the experiments (*r* ^2^ = 0.52; Figure S3B, Table S3) at this time frame for MnM-Scheme-1 complexes. This is expected, as the MnM complexes were artificially constructed and require longer simulation times to achieve stable and converged trajectories. We then increased the simulation time to 50 ns and observed the Cα RMSD for the trajectory. At the last 10 ns, the trajectories showed good convergence (Figure 4). From this time frame (40–50 ns), 100 evenly spaced frames were extracted for MM-GBSA calculations. A significant enhancement in correlation (*r* ^2^ = 0.70; Figure 5, Table S4) was observed compared to simulations starting from the crystal structures. This highlights the importance of effective conformational sampling by the MnM-Scheme-1 protocol in generating reliable starting structures for MM-GBSA calculations. Although normal mode entropy (NME) and/or replica exchange molecular dynamics (REMD) could have been done in this scenario to sample conformational phase space and account for entropic contributions, these are extremely expensive^17,101^ compared to the MnM approach applied here. Particularly, NME and REMD are not practically applicable when evaluating larger systems like PPIs and for many PPI complexes.^34,102^ Our approach will thus be beneficial for routine RBFE analysis as well as SAR analysis of peptide therapeutics.

**Figure 3:**
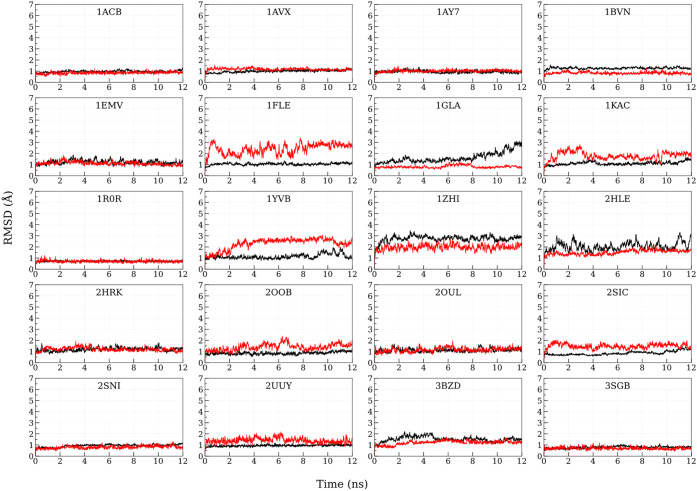
C-alpha RMSD of the individual chains (extracted from the corresponding crystal structures) for the first 12 ns. Black and red denote chains A and B, respectively (Chains are color coded in Figure 1). The time scale on the *X*-axis is given in nanoseconds (ns), and the RMSD on the *Y*-axis is expressed in angstroms for all the PPIs.

**Figure 4:**
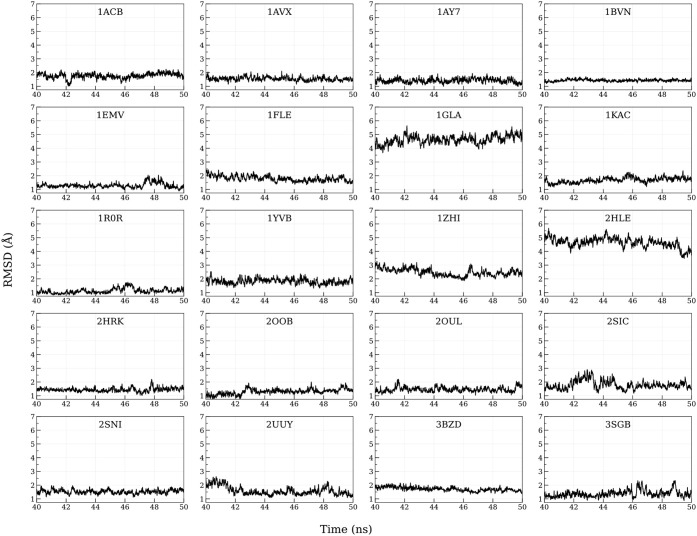
C-alpha RMSD of the PPI complexes generated using MnM-Scheme-1 for 40 to 50 ns. The time scale on the *X*-axis is given in nanoseconds (ns), and the RMSD on the *Y*-axis is expressed in angstroms for all the PPIs.

**Figure 5:**
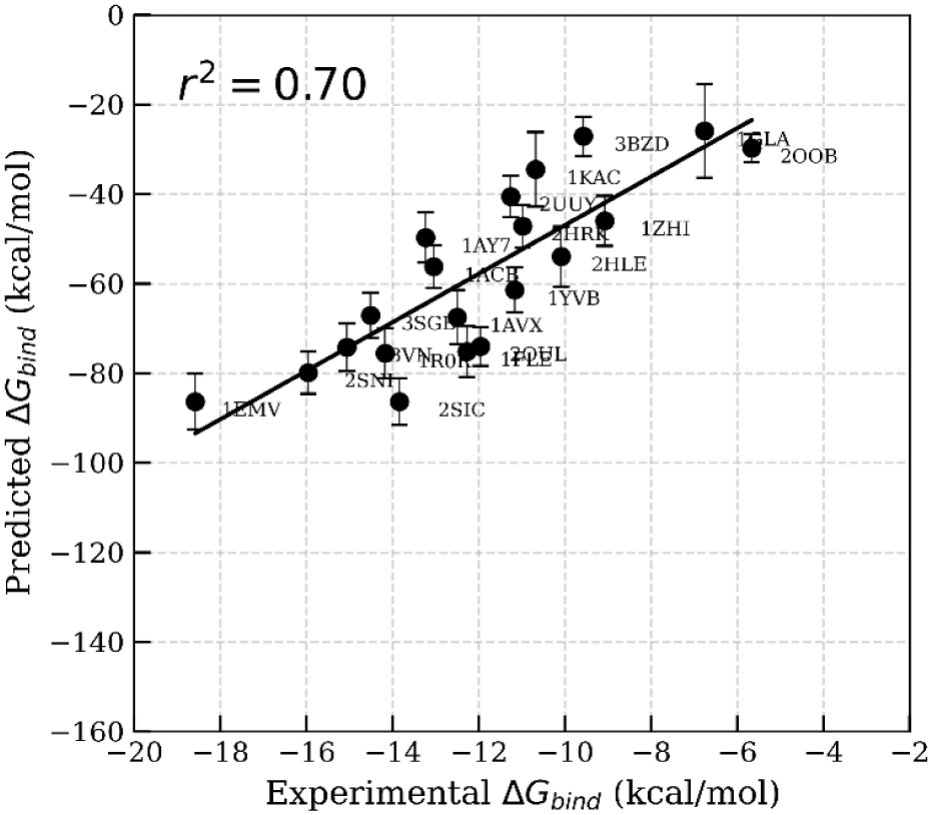
Correlation between experimental Δ*G*_bind_ and predicted binding free energies calculated using MM-GBSA. The binding free energies were calculated from 100 frames extracted at 40 to 50 ns of the MD trajectories without interfacial waters, where the starting structures originated from the MnM-Scheme-1.

### 3.2 Investigations of MnM Approach’s Impact on Key Interfacial Residues

The impact of conformational sampling through MnM is investigated to provide a comparative analysis of how this approach improves MM-GBSA estimation as well as its correlation to experimental results . We observed a significant difference in MM-GBSA predicted values between Scheme-0 and MnM-Scheme-1. In the regular approach (Scheme-0), MM-GBSA values were severely underestimated for complexes 1EMV and 1ZHI. The absolute difference in MM-GBSA values is 15.8 and 12.2 kcal/mol for complexes 1EMV and 1ZHI, respectively (Figure 6). Figure S4 illustrates the comparison of MM-GBSA values between Scheme-0 and MnM-Scheme-1 across all 20 PPI systems, excluding the inclusion of interfacial waters. The MM-GBSA values were further analyzed using pairwise energy decomposition for Scheme-0 vs MnM-Scheme-1. In 1ZHI, the MM-GBSA value was −33.7 kcal/mol in Scheme-0, while in the MnM-Scheme-1, the MM-GBSA value was −46.0 kcal/mol. The pairwise energy decomposition revealed that GLU53:ARG238 was the most impacted pair, contributing to −5.6 kcal/mol difference in MnM-Scheme-1 compared to Scheme-0 (Table S5). The frames were clustered, and the representative structure was analyzed using the clustering protocol mentioned in the Section 2.3. This reveals that MnM-Scheme-1 samples unique binding modes of the PPI complex that cannot be captured in simulations initiated from the crystal structures (Figure 7A). The atom pair distance between the OE2 atom of GLU53 in chain A and the HH11 atom of ARG238 in chain B was 6.06 Å in Scheme-0, whereas in MnM-Scheme-1, these atoms were more oriented closer to each other at a 3.14 Å distance (Figure 7C,D). This difference in the atom pair distance was consistent across all frames extracted for the MM-GBSA calculation (Figure 7B), emphasizing the strength of the MnM approach in sampling binding poses not observed in crystal structures. A similar scenario was observed for the top contributing pair LYS97:GLU170 of 1EMV, which accounted for a −11.1 kcal/mol difference (Table S5) out of a total of −15.8 kcal/mol (−70.6 kcal/mol in Scheme-0 vs −86.3 kcal/mol in MnM-Scheme-1, as shown in Figure S4). The atom pair distance between the HZ3 atom of LYS97 in chain A and the OE1 atom of GLU170 in chain B was 3.32 Å in Scheme-0, while in MnM-Scheme-1 these atoms were oriented closer to each other at a distance of 2.00 Å (Figures S5A, S5C, S5D). Moreover, this difference in the atom pair distance was also observed throughout the frames extracted for MM-GBSA analysis (Figure S5B).

**Figure 6:**
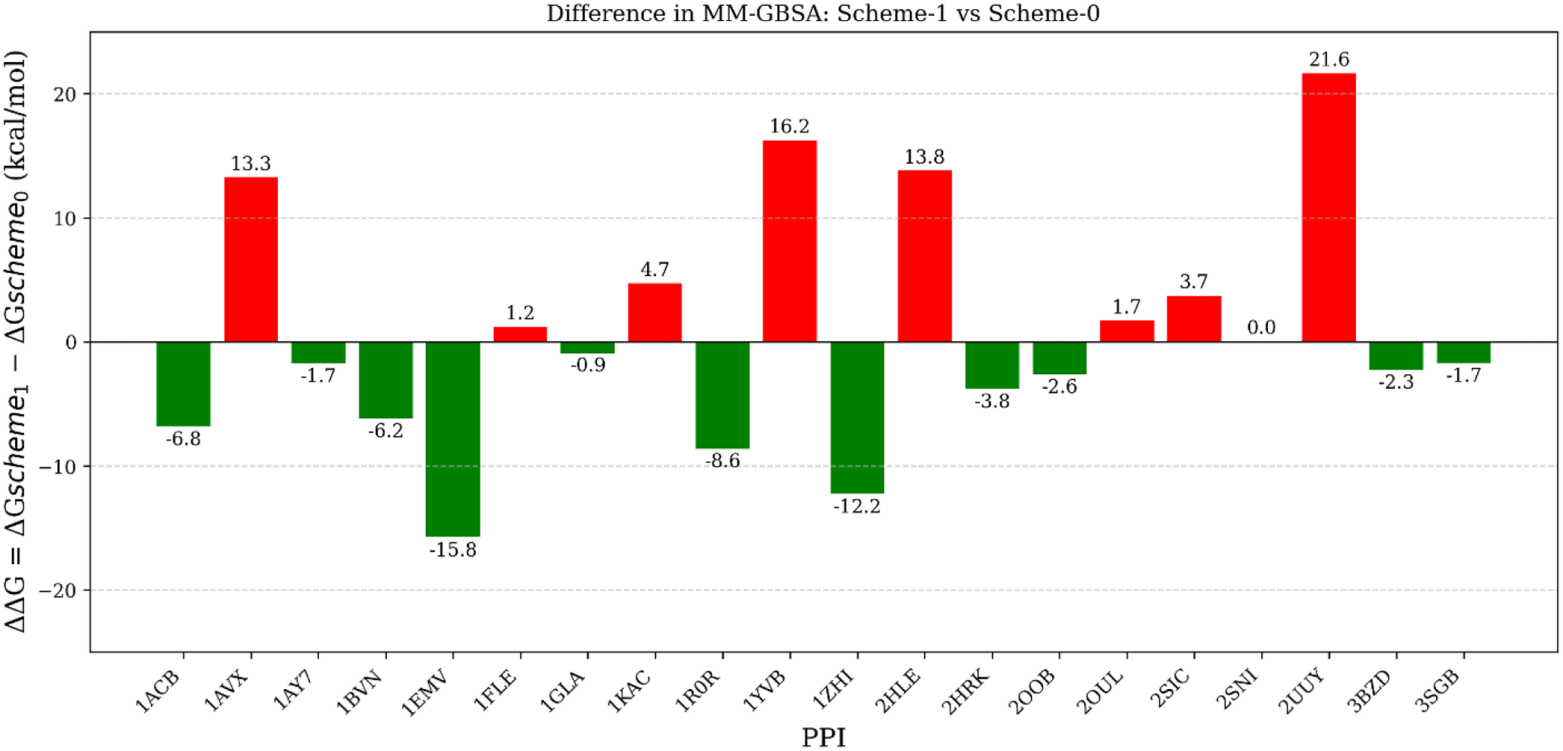
Difference in MM-GBSA values between MnM-Scheme-1 and Scheme-0 simulations (MnM-Scheme-1 – Scheme-0 MM-GBSA). Red bars represent positive differences, where Scheme-0 is overestimated, and green bars represent negative differences where Scheme-0 underestimated MM-GBSA numbers compared to the MnM-Scheme-1.

**Figure 7:**
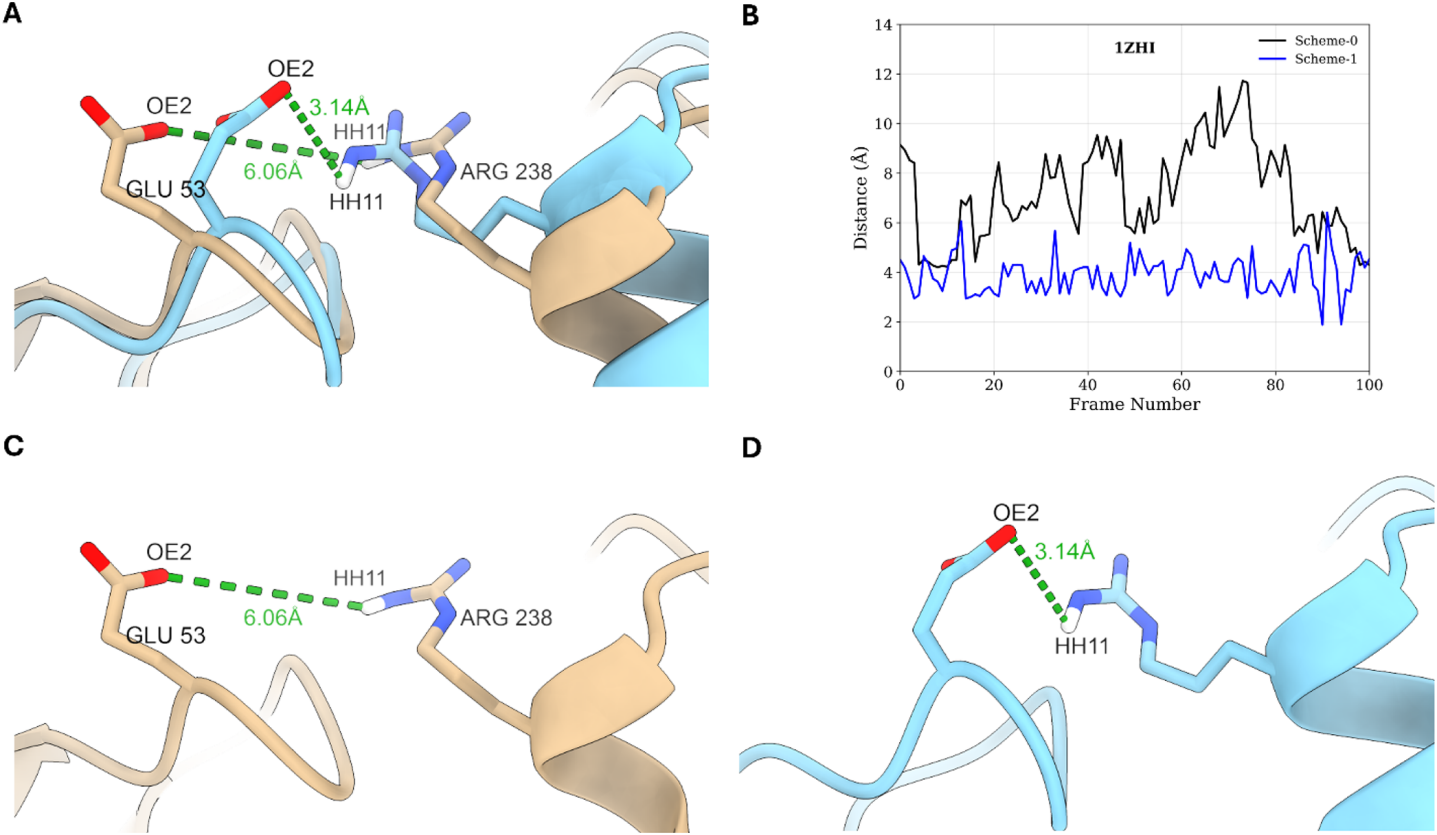
(A) Overlay of the representative structures of 1ZHI from Scheme-0 (beige) to MnM-Scheme-1 40 to 50 ns (cyan). (B) Distance between atom pairs of GLU53 OE2 and ARG238 HH11 in the 100 extracted frames for MM-GBSA calculation. (C and D) Distance between atom pairs of GLU53 OE2 and ARG238 HH11 in the representative structure of Scheme-0 and MnM-Scheme-1, respectively.

MnM protocol was also able to correct the MM-GBSA overestimation done by Scheme-0 in 1AVX, 1YVB, 2HLE and 2UUY complexes. For 1AVX, the MM-GBSA value was overestimated in Scheme-0 as −80.8 kcal/mol, whereas in MnM-Scheme-1, this overestimation was adjusted to −67.5 kcal/mol (Figure S4). The pairwise energy decomposition revealed that the ARG288:HIE23 pair contributed the most (5.2 kcal/mol difference) in MnM-Scheme-1 vs Scheme-0 (Table S5). This difference was due to the rearrangement of the side chains of HIE23 in chain A, where it was observed in a “flipped” conformation in the structures of MnM-Scheme-1 vs Scheme-0 (Figure 8A). The atom pair distance between the HD2 atom of HIE23 in chain A and the NH1 atom of ARG288 in chain B was 3.66 Å in Scheme-0, while in MnM-Scheme-1, this distance was 7.68 Å (Figure 8C vs D). This “flip” in the side chain of histidine resulted in an improved correlation of the MM-GBSA value to the experimental Δ*G* through the MnM approach, which is not observed if the simulation was started from the crystal structures. Atom pair distances from the extracted frames were also consistent in the trajectory (Figure 8B). For other complexes where the MM-GBSA was overestimated in Scheme-0 (absolute differences of 16.3 kcal/mol in 1YVB, 13.8 kcal/mol in 2HLE, and 21.7 kcal/mol in 2UUY in Figure 6), a similar trend of increased atom pair distance was observed (Figures S6–S8, respectively).

**Figure 8:**
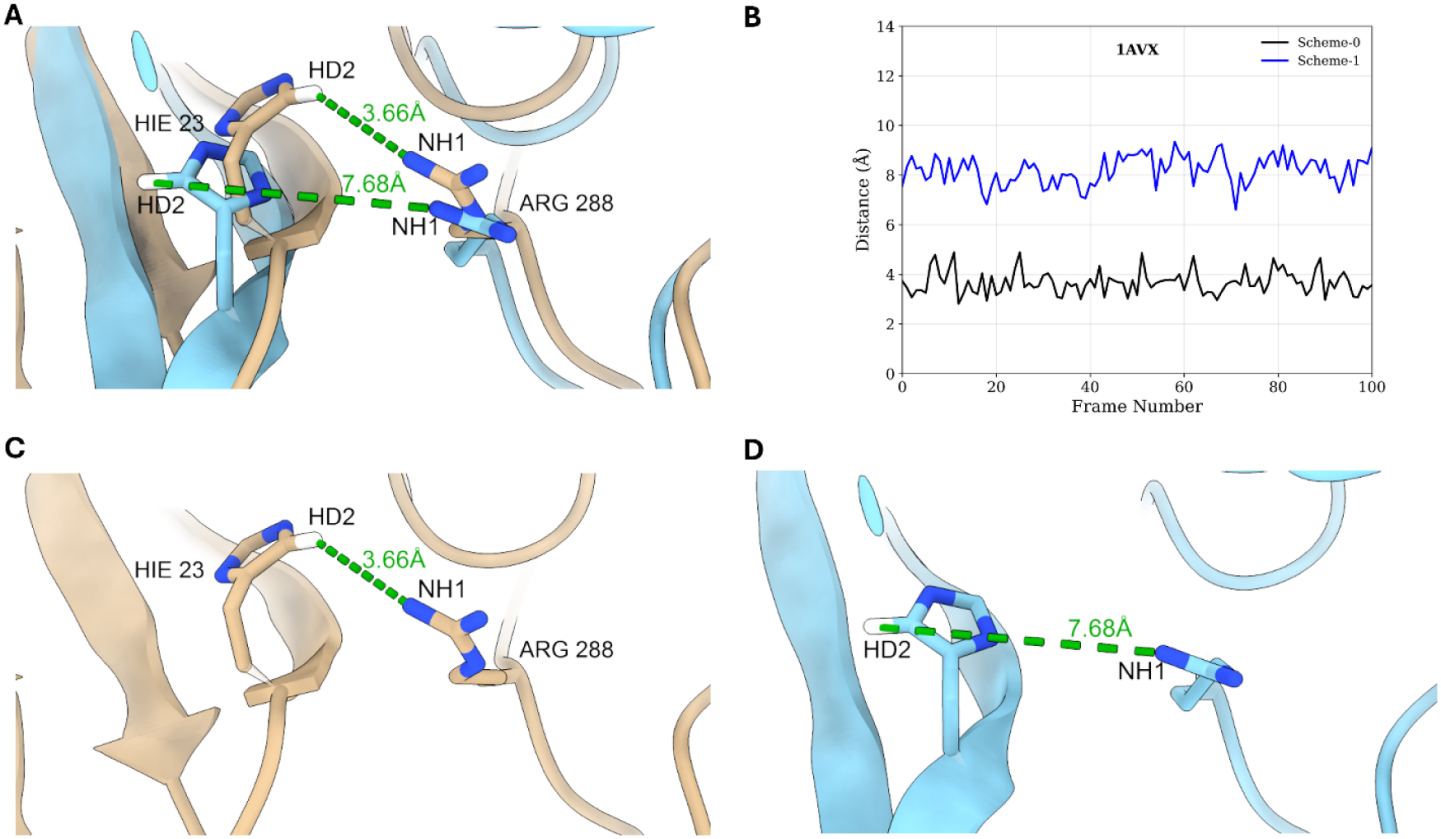
(A) Overlay of the representative structures of 1AVX from Scheme-0 (beige) vs MnM-Scheme-1 40 to 50 ns (cyan). (B) Distance between atom pairs of HIE23 HD2 and ARG288 NH1 in the 100 extracted frames for MM-GBSA calculation. (C and D) Distance between atom pairs of HIE23 HD2 and ARG288 NH1 in the representative structure of Scheme-0 and MnM-Scheme-1, respectively.

### 3.3 Inclusion of 30 Interfacial Water Molecules

The inclusion of a fixed number of interfacial waters as part of the receptor in MM-GBSA calculations showed significant improvements in binding free energy correlation as observed in previous studies.^56,58^ We thus explored the relative improvement in performance of MM-GBSA predictions upon the inclusion of 30 interfacial water molecules in “crystal structures” versus ″MnM-generated starting structures”. On the trajectories starting from crystal structures, upon including 30 interfacial waters, the correlation significantly improved (*r* ^2^ = 0.74; Figure S9A, Table S2) compared to results without interfacial waters (*r* ^2^ = 0.47; Figure S3A). This reinforces the effectiveness of incorporating interfacial waters in MM-GBSA calculations for predicting protein–protein binding free energies on a relative scale. We also investigated whether including the same number of waters improves the correlation for MnM-Scheme-1. Inclusion of 30 interfacial waters at the 4th ns of MnM-Scheme-1 starting complexes showed poor improvement in correlation. The correlation was 0.52 before the inclusion of 30 waters, as shown in Figure S3B, Table S3 and 0.60 after the inclusion of 30 waters, as shown in Figure S9B, Table S3. This occurs due to the instability of MD trajectories within a short period of time for artificially generated complexes, as previously observed. On the other hand, when the same approach was applied to the stable 40–50 ns time frame, a significant improvement in correlation was observed. The correlation increased from *r* ^2^ = 0.70 to *r* ^2^ = 0.86 (Figure 9; Table S4), which is the best correlation among all. This highlights the effect of conformational sampling applied within the MnM-W-MMGBSA approach in predicting protein–protein relative binding free energies.

**Figure 9:**
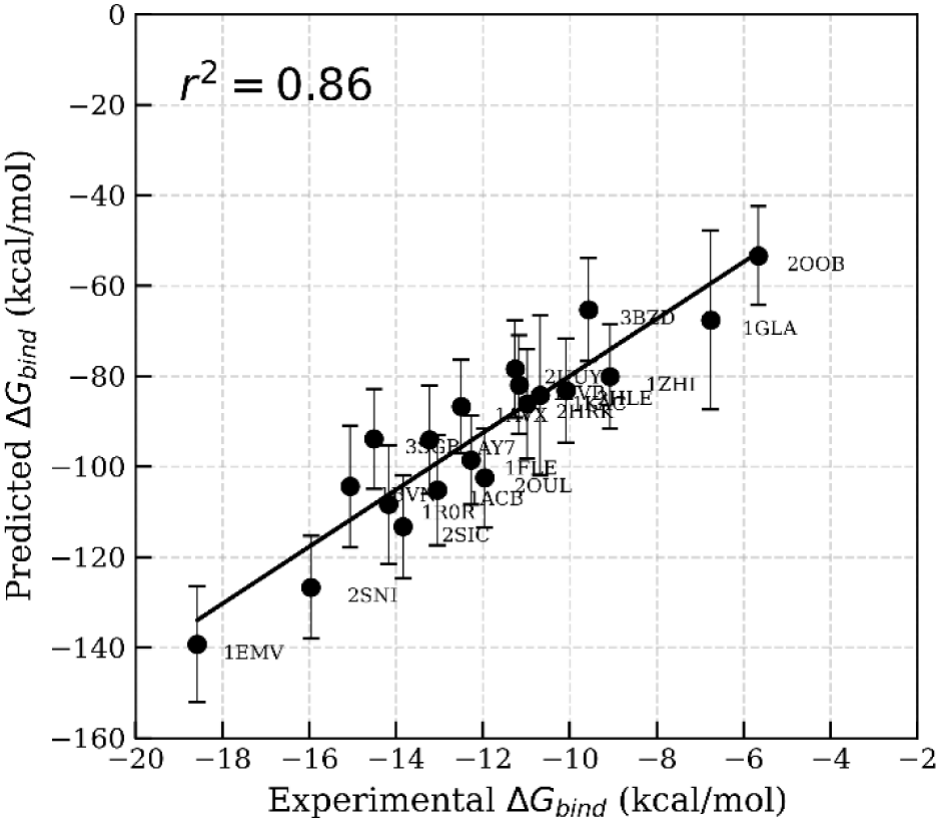
Correlation between experimental Δ*G*_bind_ and predicted binding free energies calculated using MM-GBSA. The binding free energies were calculated from 100 frames extracted at 40 to 50 ns of the MD trajectories including 30 interfacial waters, where the starting structures originated from the MnM-Scheme-1.

### 3.4 Impact on Size and Affinity of PPI

PPI complexes have variable sizes and contact surface areas, which range from 1500 to 3000 Å^2^.^103,104^ The selected 15 PPIs in this report have variable numbers of total residues and surface areas ranging from 12214 to 65639 Å^2^ (Table 3). Also, they exhibit a wide range of binding affinities (−5.7 to −18.6 kcal/mol, Table 1). To explore whether the MnM-W-MMGBSA is dependent on specific sizes and/or affinities of PPIs, we analyzed an RBFE comparison (ΔΔ*G*) of them. In both cases—without and with interfacial waters—similar correlations with Δ*G* were observed on the best-performing MnM time frame (40–50 ns of Scheme 1). Regular MM-GBSA showed a correlation of 0.70, while it also showed a similar trend in ΔΔ*G* (*r* ^2^ = 0.69, Figure 10A). Furthermore, including 30 interfacial waters, the correlations were comparable (*r* ^2^ = 0.86 for ΔΔ*G* in Figure 10B) to the Δ*G*. This signifies the MnM-W-MMGBSA protocol’s applicability to a versatile set of PPI systems with a wide range of surface areas and binding affinities.

**Table 3:** Total Number of Residues in the Selected PPI Complexes (Rec_res = Receptor Residues; Lig_res = Ligand Residues)^a^

PDB ID	Total residues	rec_res	lig_res	SASA (Å^2^)
1ACB	306	1-243	244-306	30576.22
1AVX	400	1-223	224-400	42857.52
1AY7	185	1-96	97-185	18971.09
1BVN	567	1-496	497-567	55934.68
1EMV	214	1-131	132-214	21778.30
1FLE	287	1-240	241-287	27987.42
1GLA	646	1-497	498-646	65639.54
1KAC	309	1-185	186-309	30186.50
1R0R	325	1-274	275-325	29868.04
1YVB	352	1-241	242-352	35183.93
1ZHI	333	1-207	208-333	37788.75
2HLE	326	1-188	189-326	38230.64
2HRK	298	1-177	178-298	34068.59
2OOB	116	1-72	73-116	12214.00
2OUL	348	1-241	242-348	35409.68
2SIC	382	1-275	276-382	34612.92
2SNI	339	1-275	276-339	31720.70
2UUY	275	1-223	224-275	26449.46
3BZD	343	1-234	235-343	34601.45
3SGB	235	1-185	186-235	21732.75

a The solvent accessible surface area (SASA) was calculated using PyMOL.

**Figure 10:**
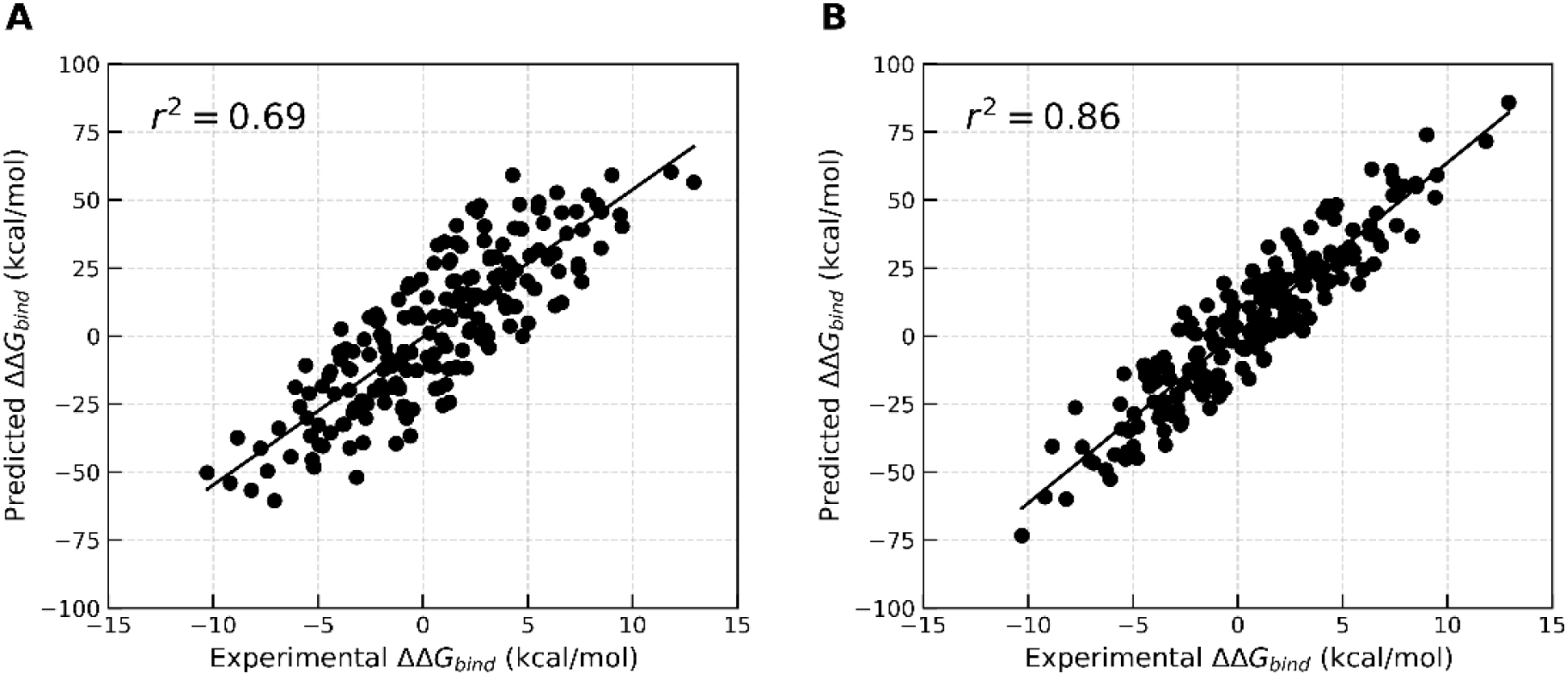
Comparison of the difference in relative binding free energy (ΔΔ*G*) between experimental and predicted. MM-GBSA was calculated from 100 frames extracted at 40 to 50 ns of the MD trajectories, where the starting structures originated from the MnM approach (MnM-Scheme-1) without incorporating interfacial waters (A) and including 30 interfacial waters (B).

### 3.5 Simulation Time for Generating MnM

We explored the optimum simulation length to sample the individual chains for the MnM strategy. For that, an alternative MnM simulation protocol (MnM-Scheme-2) was performed, in which individual chains were simulated for 100 ns. The representative structures were taken from the last 20 ns (80–100 ns) time frame to generate starting geometries of the selected PPI complexes. These complexes were then subjected to 50 ns MD simulations following the same protocol used in MnM-Scheme-1. MM-GBSA calculations were performed on the last 10 ns (40–50 ns). The correlation (*r* ^2^) values for these time frames were 0.00 (Figure S10A) and 0.01 (Figure S10C) without the interfacial waters for the 4th ns and 40–50 ns, respectively, and 0.02 (Figure S10B) and 0.03 (Figure S10D) with 30 interfacial waters for the 4th ns and 40–50 ns, respectively. The corresponding MM-GBSA values are given in Table S6 for the 4th ns and Table S7 for 40 to 50 ns of MnM-Scheme-2. These poor correlations suggest that prolonged simulations of individual chains (>12 ns) result in conformational sampling that deviates significantly from the native bound complex. This is also evident from the unstable Cα RMSD of individual chains sampled from MnM-Scheme-2 (Figure 11). In summary, MnM-Scheme-1 remains the optimal strategy for predicting PPI binding free energies, as MnM-Scheme-2 fails to sample native-like conformations.

**Figure 11:**
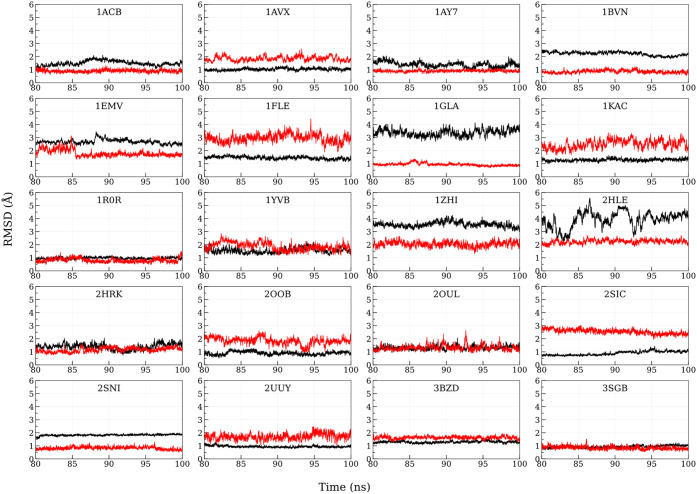
Cα RMSD of the individual chains (extracted from corresponding crystal structures) for 80 to 100 ns. Black and red denote chains A and B, respectively (Chains are color coded in Figure 1). The time scale on the *X*-axis is given in nanoseconds (ns), and the RMSD on the *Y*-axis is expressed in angstroms for all the PPIs.

### 3.6 Reproducibility and Outliers

The reproducibility of the MnM-W-MMGBSA method was assessed by running a duplicate MD simulation for the best-performing MnM-Scheme-1. MM-GBSA was calculated for the 40–50 ns time frame. The second independent run yielded reproducible results, showing correlation coefficients (*r* ^2^) of 0.69 (Figure S11A) and 0.85 (Figure S11B) with the experimental binding free energies for simulations without and with 30 explicit water molecules, respectively. In the quest to improve the correlation, we investigated possible outliers and found that the binding affinity of 3SGB is largely underestimated. Structural analysis revealed that in 3SGB, the smaller protein contains a long-disordered tail located directly at the binding interface (Figure 12A) which is often considered an unusual and challenging PPI in previous reports.^105^ It largely impacted the density of interfacial water molecules, where they are more scattered and more closely located near the receptor (Figure 12C). In contrast, for ordered PPIs the interfacial water density is more evenly distributed at the interface. An example of water density distribution for ordered PPIs like 1AY7 is presented in Figure 12B, while other PPIs are illustrated in Figure S12. Wang et al.^105^ applied a highly accurate and rigorous metadynamics-based dissociation free energy method, yet struggled to reproduce the experimental binding free energy of 3SGB. Interestingly, when this PPI is discarded from the data set, the correlation between calculated and experimental binding free energy increased to 89% (Figure 12D) for the best-performing MnM scheme and time frame (MnM-Scheme-1, 40 to 50 ns with interfacial waters). Similar improvement is also observed for crystal starting structures (*r* ^2^ = 0.76, Figure 12E). Extended studies need to be conducted for these types of PPI systems to resolve this issue, focusing on the correct identification of protonation states of titratable residues and/or exhaustive sampling of the tail portion of the smaller protein chain.

**Figure 12:**
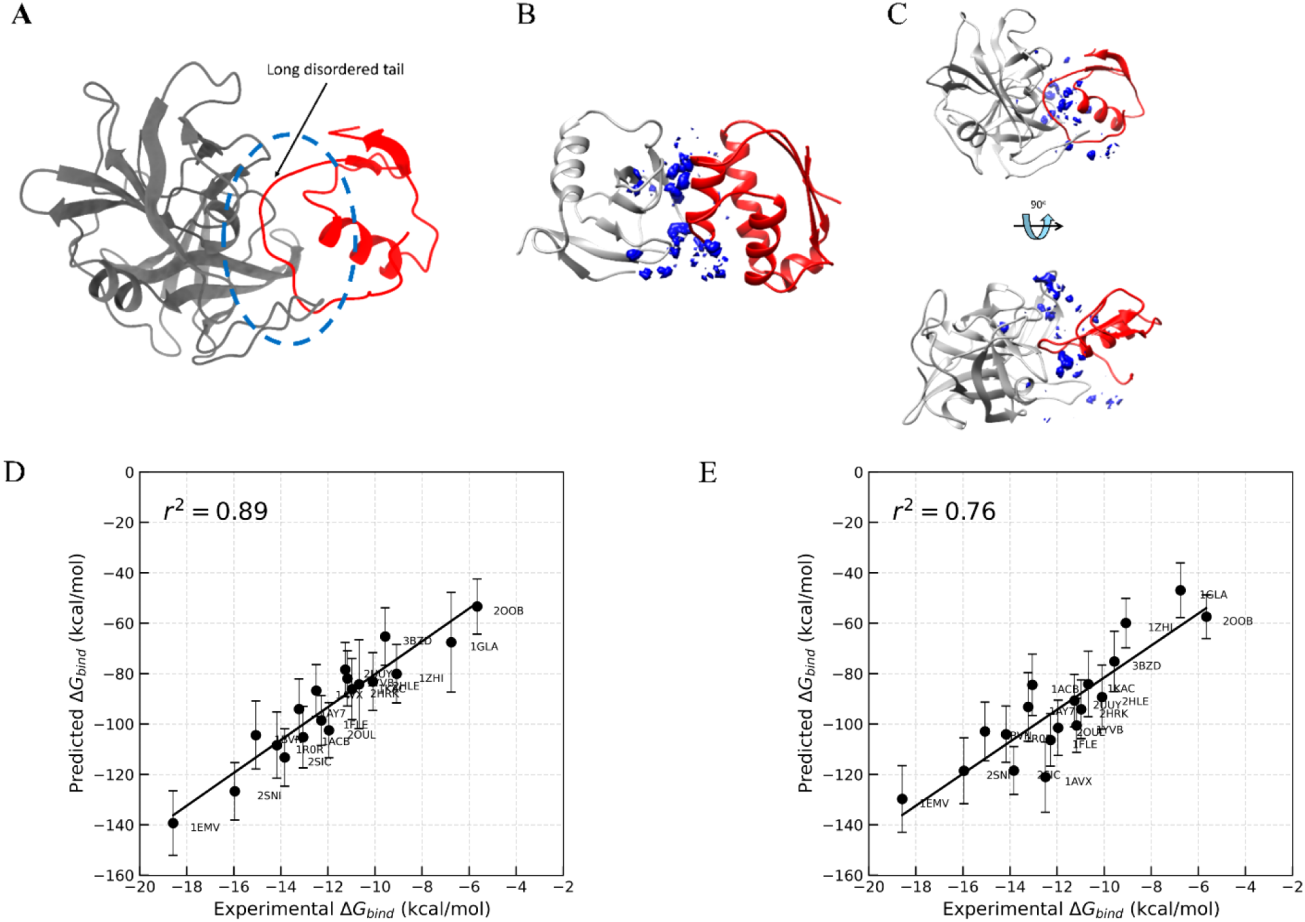
Effect of the outlier (PDB ID: 3SGB) on the correlation of MnM-W-MMGBSA to the experimental binding free energies. (A) Long disordered tail of 3SGB in the PPI binding interface highlighted in a blue dashed circle. (B) Water density plot of 1AY7 for the MnM-Scheme-1 complex at 40 to 50 ns visualized with Chimera. (C) Water density plot of 3SGB for the MnM-Scheme-1 complex at 40 to 50 ns visualized with Chimera, showing both front view and top view. (D) Correlation between experimental Δ*G*_bind_ and predicted binding free energies calculated using MM-GBSA, excluding the PPI complex 3SGB. The analysis includes 30 interfacial waters extracted from MD trajectories between 40 and 50 ns, starting from structures generated through the MnM-Scheme-1 approach and (E) at the 4th ns starting from the crystal structures.

### 3.7 Side-Chain RMSF Comparison between the Schemes

The side-chain RMSF was analyzed for the selected time frames for all three starting structures (Scheme-0, MnM-Scheme-1, and MnM-Scheme-2). For the crystal, the 4th ns time frame was used, while for MnM-Scheme-1 and MnM-Scheme-2, 40–50 ns time frames were selected to calculate the RMSF. A higher RMSF was observed for MnM-Scheme-2 starting structures compared to a lower RMSF in MnM-Scheme-1 starting structures. Upon comparing the crystal structure trajectory, it was revealed that MnM-Scheme-1 RMSF is closer to the crystal structure, while MnM-Scheme-2 deviated significantly. Figure 13 shows the side-chain RMSF for 4 of the selected PPI complexes, while the rest of the complexes are provided in Figure S13. This suggests that the MnM-Scheme-2 complexes have significant structural deviations from the crystal structure, which results in a poor correlation.

**Figure 13:**
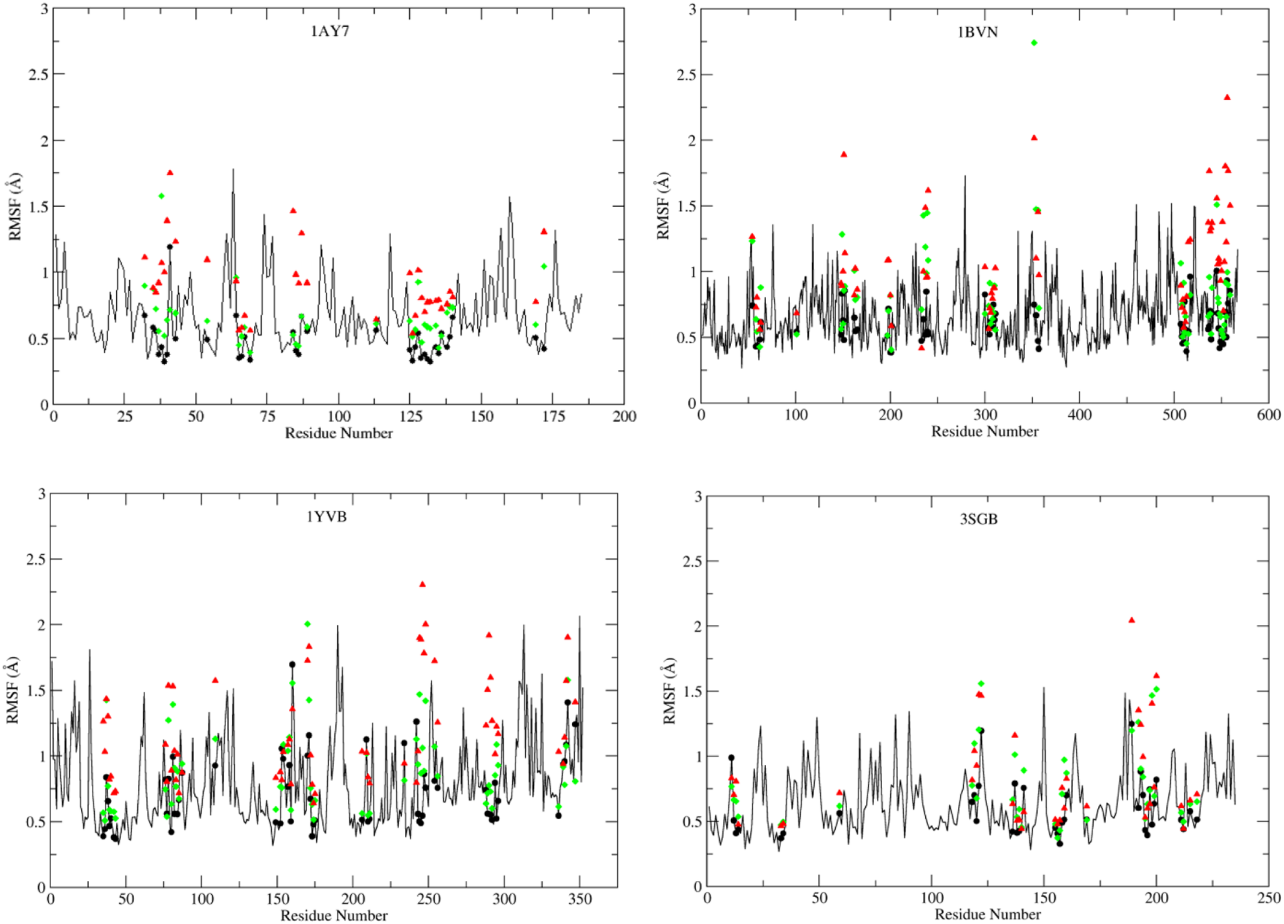
Side-chain RMSF comparison between the starting structures. The 4th-ns simulation of the crystal structures is shown by the black line, and interfacial residues are highlighted with black dots. RMSF for MnM-Scheme-1 interfacial residues (40–50 ns) is highlighted as green diamonds and that for MnM-Scheme-2 interfacial residues (40–50 ns) is highlighted as red triangles. The *X*-axis represents the residue numbers, and The *X*-axis represents the residue numbers, and the *Y*-axis shows the RMSF values.

## 4 Conclusions and Future Perspectives

Protein–protein interactions (PPIs) are fundamental to the regulation of biological systems, forming the structural and functional frameworks for numerous cellular processes. Dysregulated PPIs have been closely linked to various human diseases.^10,106^ Consequently, PPIs have emerged as a highly attractive class of therapeutic targets.^107^ Although PPIs were once considered largely undruggable, continued research has revealed that many can be effectively modulated with the right strategies.^108^ A landmark achievement in this domain is Venetoclax,^109^ a selective BCL-2 inhibitor and the first FDA-approved PPI-targeting drug, which underscores the therapeutic potential and feasibility of targeting PPIs. Within the challenging landscape of PPI drug discovery, accurately estimating binding free energy remains a critical task—especially due to the dynamic and solvent-exposed nature of PPI interfaces. In this context, we explored a modified variant of the MM-GBSA method, known as MnM-W-MMGBSA, which addresses both the challenges of conformational sampling and local solvation effects by dynamically incorporating a fixed number of interfacial water molecules throughout molecular dynamics (MD) simulations. Unlike standard MM-GBSA, this approach accounts for the effective sampling of the most populated conformations of the PPI and the transient yet functionally significant role of water molecules at PPI interfaces—especially those involved in bridging interactions or stabilizing flexible regions. These aspects are particularly crucial in PPIs, where conformational plasticity and solvent mediation are prominent. Importantly, this modified method overcomes the inability of *gmx_MMPBSA* tool to include the interfacial water molecules as part of the complex/receptor by efficiently converting GROMACS topologies into Amber topology in an automated, streamlined, and reproducible manner. Our protocol will be particularly useful in the high-throughput screening of protein–protein and protein–peptide complexes, particularly in the emerging field of peptide therapeutics.

We benchmarked MnM-W-MMGBSA for two different time frames of our Mix-matched complexes to systematically improve the correlations. Notably, MnM provides a cost-effective approach to conformational sampling that captures PPI flexibility, eliminating the need for expensive protocols such as REMD. The protocol shows 70% correlation to the experiments compared with 47% (Scheme-0) by addressing conformational sampling alone. Moreover, by including interfacial solvent, this correlation improved to 86%. We also found this protocol to be suitable for a wide range of PPIs in terms of size and affinity. Additionally, this protocol is robust and reproducible, which makes it reliable in routine peptide-based drug discovery projects. To effectively account for the conformational sampling and structural dynamics, a simulation duration of 50 ns gives the optimum correlation for the MnM complexes, which is reasonable for such large PPI systems. Based on our findings, MnM-W-MMGBSA offers a robust, efficient, and more physically meaningful alternative for estimating binding affinities in systems where conformational flexibility and solvation effects play critical roles, such as PPIs. A modified version of our Mix-and-Match method was successfully applied to investigate the binding free energy landscape of a protein–protein complex involving intrinsically disordered regions, demonstrating the method’s applicability to more complex protein–protein interactions.^59^ In future work, we plan to apply the MnM-W-MMGBSA strategy to refine peptide docking poses against protein–protein interaction (PPI) targets, particularly for designing peptide-based therapeutics against challenging PPIs. By combining docking/AlphaFold with MnM-based conformational refinement and MM-GBSA evaluation, we aim to identify native-like peptide binders with improved binding affinity. This direction will extend the impact of the current methodology toward rational peptide design and enhance its translational relevance in peptide-based inhibitor discovery for PPI targets.

## Supplementary Material



## Data Availability

The free software tools used in this study, including UCSF ChimeraX (www.cgl.ucsf.edu/chimerax/), PyMOL (pymol.org), XMGRACE (https://plasma-gate.weizmann.ac.il/Grace/), and VMD (www.ks.uiuc.edu/Research/vmd/) are freely available at their websites. The only commercial software used in this study is MOE 2022.02 (www.chemcomp.com/) for protein preparation and protonation. The MMPBSA.py is available for use with AmberTools23 (https://ambermd.org/AmberTools.php). All the input files required to reproduce the results, as well as the initial and final structures, are available for free on GitHub (https://github.com/SahaLabGitHub/MnM-W-MMGBSA/).

## Notes

Supporting Information contains the analysis of MnM schemes. SI tables contain information related to the interfacial residues calculated for MnM-W-MMGBSA. SI figures contain correlation plots, pairwise per-residue MM-GBSA analysis, as well as RMSD plots for the individual PPI explored in the schemes (DOCX)
