# Supplementary material for: MnM-W-MMGBSA: A Computational Strategy to Improve Relative Binding Free Energies of Protein–Protein Interaction Systems
Source: J Phys Chem B. 2025 Nov 30;129(49):12685–701. doi: 10.1021/acs.jpcb.5c04085 (PMC12703734; doi:10.1021/acs.jpcb.5c04085)
Supplement: Supplementary file 1 [file jp5c04085_si_001.docx]

**Supporting Information**

**MnM-W-MMGBSA: A Computational Strategy to Improve Relative Binding Free Energy of Protein-Protein Interaction Systems**

Md Nazmul Hasan^1^, Shilpa Sharma^1^, Justice Josiah Mallen^1^, Arjun Saha^1*^

^1^Department of Chemistry and Biochemistry, University of Wisconsin-Milwaukee, Milwaukee, WI, 53211, USA

*Corresponding author; email: [saha6@uwm.edu](mailto:saha6@uwm.edu)

**Supplementary Tables**

**Table S1:** Interfacial residues for each PPI complex calculated by “InterfaceResidues” PyMol script.

| **PDB ID** | **Starting structure** | **Interfacial residues** |
| --- | --- | --- |
| 1ACB | Scheme-0 | 39,40,41,42,57,58,59,94,99,143,146,147,149,150,151,171,172,175,190,191,192,193,195,213,214,  215,216,217,218,226,244,276,277,278,279,280,281,282,283,284,285,287,289,291,302,304,306 |
|  | MnM-Scheme-1 | 35,39,40,41,42,57,58,99,143,146,147,148,149,151,171,172,175,190,191,192,193,194,195,213,  214,215,216,217,218,244,245,276,277,278,279,280,281,282,283,284,285,287,289,291,301,304,305,306 |
|  | MnM-Scheme-2 | 39,40,41,42,57,58,59,99,143,146,147,148,149,150,151,172,175,190,191,192,193,194,195,213,  214,215,216,217,218,227,244,276,277,278,279,280,281,282,283,284,285,289,291,304 |
| 1AVX | Scheme-0 | 20,22,23,24,25,40,41,43,76,78,79,81,123,128,129,131,155,171,172,173,174,175,177,191,192,193,  194,195,196,197,204,205,206,224,225,233,235,236,237,238,282,283,284,285,286,287,288,289,  293,294,295,340,342,361,363,364,365,398 |
|  | MnM-Scheme-1 | 20,22,23,24,25,40,41,42,43,55,56,77,78,79,80,81,121,123,129,131,153,154,155,172,173,174,175,  177,191,192,193,194,195,196,204,206,224,225,233,235,236,237,238,239,282,283,284,285,286,  287,288,289,291,294,295,297,315,340,342,360,361,362,363,364 |
|  | MnM-Scheme-2 | 20,22,23,24,25,40,41,42,43,55,76,77,78,79,80,81,121,128,129,130,131,154,155,172,173,174,175,  177,191,192,193,194,195,204,205,224,225,233,235,236,237,238,280,282,283,284,285,286,287,  288,289,291,294,295,338,340,342,361,362,363,364 |
| 1AY7 | Scheme-0 | 32,35,36,37,38,39,40,41,43,54,64,65,66,67,69,84,85,86,87,89,113,125,126,127,128,  129,130,131,132,134,135,136,138,139,140,169,172 |
|  | MnM-Scheme-1 | 32,35,36,37,38,39,40,41,43,54,64,65,66,67,69,84,85,86,87,125,126,127,128,129,  130,131,132,134,135,136,138,139,140,165,168,169,172 |
|  | MnM-Scheme-2 | 32,35,36,37,38,40,41,43,54,64,65,66,67,69,84,85,86,89,123,125,126,127,129,130,  131,132,134,135,138,139,140,165,168,169,172,176 |
| 1BVN | Scheme-0 | 54,58,59,62,63,101,148,149,150,151,152,162,163,165,197,198,200,201,233,235,  237,238,239,240,300,304,305,306,307,308,309,310,311,352,354,356,357,507,  508,509,510,511,512,513,515,517,536,537,538,539,540,545,546,547,548,549,  550,551,552,553,554,555,556,557,559 |
|  | MnM-Scheme-1 | 58,59,62,63,98,148,149,150,151,152,156,162,163,164,165,195,197,198,200,201,233,  235,237,238,239,240,256,299,300,303,304,305,306,308,309,310,311,352,356,357,507,  508,509,510,511,512,513,515,517,537,538,539,540,541,542,545,546,547,548,550,551,  552,553,554,555,556 |
|  | MnM-Scheme-2 | 58,59,62,63,98,101,148,149,150,151,152,153,156,162,163,164,165,197,198,200,201,  235,236,237,257,260,261,300,303,304,305,306,307,308,309,353,357,508,509,510,511,  512,513,515,517,530,536,537,538,539,540,544,545,546,547,548,549,550,551,552,553,  554,555,556,557,559 |
| 1EMV | Scheme-0 | 54,70,71,72,73,74,75,77,78,81,83,84,86,87,88,89,90,92,95,97,98,99,151,152,153,154,  156,157,158,159,162,163,166,167,170,176,177,178,179,180,182,183,184,185,191 |
|  | MnM-Scheme-1 | 23,54,70,71,72,73,74,75,77,78,81,83,84,86,87,88,89,90,92,95,96,97,98,99,151,152,153,  154,155,156,157,158,159,160,162,163,166,167,170,175,176,177,178,179,180,182,183,  184,185,191 |
|  | MnM-Scheme-2 | 23,54,55,70,71,72,73,74,75,77,78,81,83,84,86,87,88,89,92,94,95,96,97,98,99,100,151,  152,153,154,155,156,157,158,159,160,162,163,164,166,167,170,176,179,180,182,183,  184,185,191 |
| 1FLE | Scheme-0 | 20,29,30,45,48,49,84,85,86,87,88,89,134,141,164,167,184,185,186,188,206,207,208,  209,210,211,212,219,247,248,249,250,251,252,253,254,255,256,257,259,261,276,277,  278,279,282,283,284 |
|  | MnM-Scheme-1 | 20,28,29,45,46,48,49,50,83,85,86,88,89,132,141,164,167,184,185,186,188,206,207,208,  209,211,212,219,248,249,250,251,252,253,254,255,256,257,259,261,277,278,279,282,  283,284 |
|  | MnM-Scheme-2 | 20,28,29,30,45,46,48,49,85,86,87,88,89,141,164,167,184,185,186,188,206,207,208,  209,211,212,219,248,249,250,251,252,253,254,255,256,257,259,261,277,278,279,  280,282 |
| 1GLA | Scheme-0 | 335,336,400,425,469,470,471,472,473,474,475,476,477,478,479,482,517,518,519,520,  522,524,525,548,549,550,551,554,567,569,572,573,575,576,578,588,611 |
|  | MnM-Scheme-1 | 335,336,387,400,424,425,426,469,470,471,472,473,474,475,476,477,478,479,480,482,  516,517,518,519,520,524,525,548,550,551,552,554,567,569,571,572,573,575,576,578,611,623 |
|  | MnM-Scheme-2 | 333,334,335,336,338,339,341,362,363,364,365,366,367,368,370,373,376,384,387,418,419,  422,423,425,470,471,472,473,474,475,476,477,478,479,480,481,482,483,485,486,487,490,513,  515,516,517,518,519,520,521,522,523,524,525,526,547,548,549,550,551,552,553,554,557,559,565,  567,569,571,572,573,575,576,578,579,580,581,582,583,584,588,609,610,611,614,616,622 |
| 1KAC | Scheme-0 | 11,13,14,15,16,23,24,27,48,49,53,83,85,86,87,92,94,95,96,97,98,213,215,216,217,  219,221,223,233,234,235,238,240,241,243,245,246,247,248,250,286,288,290,291,  292,293 |
|  | MnM-Scheme-1 | 1,11,13,14,15,16,23,24,46,47,48,49,53,84,85,86,87,92,94,95,96,98,171,173,213,215,  216,217,219,221,223,231,235,238,240,241,245,248,250,286,288,290,291,293,295 |
|  | MnM-Scheme-2 | 11,13,14,15,16,23,24,27,46,48,49,53,83,84,85,86,87,92,94,95,96,98,213,215,216,217,  219,221,223,233,234,235,238,240,241,243,245,246,247,248,250,286,288,290,291,293 |
| 1R0R | Scheme-0 | 33,61,63,95,96,98,99,100,101,102,103,106,124,125,126,127,128,151,153,154,187,188,  208,216,217,218,219,220,221,279,280,282,283,284,285,286,287,288,289,290,301,304,324 |
|  | MnM-Scheme-1 | 32,33,61,63,95,96,98,99,100,101,102,103,124,125,126,127,128,151,153,154,187,188,  208,215,216,217,219,220,276,279,280,282,283,284,285,286,287,288,289,290,291,301,  302,303,304,305,308,324 |
|  | MnM-Scheme-2 | 51,61,63,95,96,97,98,99,100,101,102,103,104,124,125,126,127,128,154,187,188,208,  216,217,218,220,279,280,282,283,284,285,286,287,288,289,290,291,301,302,303,304,  305,308,324 |
| 1YVB | Scheme-0 | 35,36,37,38,39,40,42,43,76,77,78,80,81,82,83,84,85,87,109,149,152,153,154,157,158,  159,160,170,171,172,173,174,175,206,209,210,211,234,242,243,244,245,246,247,248,  254,256,288,289,290,291,292,294,295,296,336,339,340,341,342,347 |
|  | MnM-Scheme-1 | 35,36,37,38,39,40,42,43,78,80,81,82,83,84,109,149,152,153,154,157,158,159,160,171,  172,173,174,175,206,210,211,242,243,244,245,246,247,248,256,259,288,289,290,291,  292,293,294,295,296,297,336,339,340,345,347 |
|  | MnM-Scheme-2 | 33,34,35,36,37,38,39,40,42,43,78,80,81,82,152,153,154,157,158,160,171,173,174,206,  207,208,209,210,242,243,244,245,246,247,248,254,255,256,259,288,289,290,291,292,  293,294,295,296,336,339,340,341,342,347 |
| 1ZHI | Scheme-0 | 53,54,55,56,57,58,84,85,86,88,90,108,109,112,113,115,116,117,121,160,209,210,211,212,  213,214,215,226,227,228,229,230,238,241,242,246,247,250,279,281 |
|  | MnM-Scheme-1 | 51,52,53,54,55,56,57,85,86,87,88,89,90,109,112,113,115,116,121,160,210,211,214,215,225,  226,227,228,229,238,242,279,281 |
|  | MnM-Scheme-2 | 54,55,56,57,58,84,85,86,87,88,89,90,109,112,113,115,116,121,160,209,210,211,213,214,  215,226,227,228,229,241,242,281 |
| 2HLE | Scheme-0 | 19,20,21,31,35,36,37,38,39,40,41,42,44,45,46,48,49,51,53,85,87,92,93,139,141,142,143,144,  147,148,149,150,151,176,177,178,180,218,220,257,258,259,260,261,264,267,268,269,270,271,  272,274,276,277,278,279,280,281,282,283,284,285,286,288 |
|  | MnM-Scheme-1 | 9,20,21,35,36,37,38,39,40,41,42,44,45,46,47,48,49,51,53,56,83,85,87,92,93,94,139,141,143,  144,147,148,149,151,177,178,180,218,220,257,258,259,260,261,267,268,269,270,271,272,  274,276,277,278,279,280,281,282,283,284,285,286,288 |
|  | MnM-Scheme-2 | 17,19,20,21,35,36,37,38,39,40,41,42,44,45,46,48,49,51,53,85,86,87,91,92,139,141,146,147,  148,149,150,151,177,178,180,216,218,255,256,257,258,259,260,261,267,268,269,270,271,272,  273,274,276,277,278,279,280,281,282,283,284,285,286,288 |
| 2HRK | Scheme-0 | 101,105,106,107,108,109,112,113,114,140,143,144,146,147,150,151,152,154,155,156,  228,229,230,231,232,233,236,237,238,239,269,273,274,276,277,278,280,281,284,285,  296,298 |
|  | MnM-Scheme-1 | 102,105,106,107,108,109,112,113,114,115,140,143,144,146,147,150,151,154,155,195,  201,228,229,230,231,232,233,236,237,238,239,269,273,274,276,277,278,280,281,284,  285,287,296,297,298 |
|  | MnM-Scheme-2 | 104,105,106,107,108,109,112,113,114,139,140,143,144,146,147,150,151,154,155,156,  229,230,231,232,233,236,237,238,239,269,270,273,274,276,277,280,281,284,285,296,  297,298 |
| 2OOB | Scheme-0 | 6,8,42,44,45,46,47,48,49,66,68,69,70,75,76,77,78,80,81,82,84,85,90,94 |
|  | MnM-Scheme-1 | 6,8,42,44,45,46,47,48,49,68,70,71,72,75,76,77,78,80,81,82,84,85,87,90,94 |
|  | MnM-Scheme-2 | 6,8,42,44,45,46,47,48,49,66,68,69,70,71,72,75,76,77,78,80,81,82,84,85,89,90,91,94 |
| 2OUL | Scheme-0 | 35,36,37,38,39,40,42,43,76,77,78,80,81,82,83,84,85,87,149,152,153,154,157,158,170,  171,172,173,174,175,206,209,210,234,267,268,269,270,271,272,273,275,279,281,293,  294,295,297,298,300,301,302,303,304,305,327,329,330,331,339 |
|  | MnM-Scheme-1 | 35,36,37,38,40,42,43,78,80,81,82,83,84,85,149,152,153,154,157,158,159,160,170,171,  172,173,174,175,206,209,210,267,268,269,270,271,272,273,275,281,293,295,297,298,  300,301,302,303,304,305,306,309,327,329,330,331,332,337 |
|  | MnM-Scheme-2 | 35,36,37,38,39,40,42,43,76,78,80,81,82,83,84,85,87,149,152,153,154,157,158,160,170,  171,172,173,174,175,206,209,210,234,267,268,269,270,271,272,273,275,281,293,295,  297,298,300,301,302,303,304,305,306,327,329,330,331,332 |
| 2SIC | Scheme-0 | 62,64,96,99,100,101,102,103,104,107,125,126,127,128,129,130,131,135,152,154,155,  156,167,168,188,189,217,218,219,220,221,222,334,336,337,338,339,340,341,342,343,  344,367,370,371,373 |
|  | MnM-Scheme-1 | 32,62,64,96,99,100,101,102,103,104,107,126,127,128,129,130,135,154,155,156,167,168,  188,189,217,218,219,220,221,222,289,334,335,336,337,338,339,340,341,342,343,344,345,  367,369,370,371,373 |
|  | MnM-Scheme-2 | 62,64,96,99,100,101,102,103,104,107,125,126,127,128,129,130,131,132,152,154,155,  156,167,188,189,216,217,218,219,220,221,222,289,290,334,336,337,338,339,340,341,  342,343,344,366,367,369,370,371,375 |
| 2SNI | Scheme-0 | 33,62,63,64,96,99,100,101,102,103,104,107,125,126,127,128,129,135,152,154,155,156,  167,168,189,217,218,219,220,221,222,305,306,307,308,309,310,311,312,313,314,315,  316,317,318,323,334,337 |
|  | MnM-Scheme-1 | 62,64,96,99,100,101,102,103,104,107,125,126,127,128,129,152,154,155,156,157,189,  217,218,219,221,222,305,306,307,308,309,310,311,312,313,314,315,316,317,318,319,  321,323,334,337,339 |
|  | MnM-Scheme-2 | 1,32,62,64,96,99,100,101,102,103,104,107,125,126,127,128,129,130,155,156,159,167,  168,189,216,217,218,219,221,222,305,307,308,309,310,311,312,313,314,315,316,317,  318,319,321,323,325,334,337,338,339 |
| 2UUY | Scheme-0 | 22,23,24,25,40,41,42,43,55,81,121,128,129,131,155,171,172,173,174,175,177,191,192,  193,194,195,195,196,197,204,205,235,236,237,238,239,240,241,242,243,256,257,258 |
|  | MnM-Scheme-1 | 22,23,24,25,40,42,43,78,81,84,121,123,128,129,131,155,172,173,174,175,177,191,192,  193,194,195,204,206,235,236,237,238,239,240,241,242,243,256,257,258,260,265 |
|  | MnM-Scheme-2 | 20,21,22,23,24,25,40,41,42,43,55,78,81,84,121,131,152,173,174,175,177,191,192,193,  194,195,196,235,236,237,238,239,240,241,242,243,256,257,258,260,265 |
| 3BZD | Scheme-0 | 17,18,19,22,25,57,58,59,89,90,99,100,101,103,173,174,203,204,205,207,261,263,280,283,  284,285,286,287,288,289,290,298,299,300,302,303,304,305,306 |
|  | MnM-Scheme-1 | 17,18,19,21,22,24,25,28,57,58,59,89,90,101,102,103,105,172,173,174,203,204,205,207,  261,262,263,280,283,284,285,286,287,288,289,290,294,298,299,300,302,303,304,305,306 |
|  | MnM-Scheme-2 | 17,18,19,22,25,57,58,59,89,90,101,102,103,172,173,174,203,207,260,261,262,263,280,283,  284,285,286,287,288,289,290,294,295,296,298,299,300,301,302,303,304,305,306,313 |
| 3SGB | Scheme-0 | 11,12,13,14,33,34,59,118,119,120,121,122,136,137,138,139,140,141,155,156,157,158,159,  160,169,189,192,193,194,195,196,197,198,199,200,211,212,215,218 |
|  | MnM-Scheme-1 | 10,11,12,13,14,33,59,118,119,120,121,122,136,137,138,139,140,141,155,156,157,158,159,  161,169,189,192,193,194,195,196,197,198,199,200,208,211,212,214,215,218 |
|  | MnM-Scheme-2 | 10,11,12,13,14,33,34,41,47,103,117,118,119,120,121,122,136,137,138,139,141,157,158,159,  161,169,192,193,194,195,196,197,198,199,200,211,212,214,215,218 |

**Table S2.** MMGBSA results obtained from the 4^th^ ns of MD Simulation for starting structures originated from the X-ray crystal structures obtained from RCSB PDB (Scheme-0).

| **PDB_ID** | **Experimental_dG** | **No_interfacial_water** | | **30_interfacial_waters** | |
| --- | --- | --- | --- | --- | --- |
|  |  | **∆G_bind_** | **Std. Dv.** | **∆G_bind_** | **Std. Dv.** |
| 1ACB | -13.05 | -49.35 | 4.21 | -84.45 | 12.19 |
| 1AVX | -12.50 | -80.78 | 7.733 | -121.05 | 13.96 |
| 1AY7 | -13.23 | -47.97 | 4.27 | -93.19 | 13.63 |
| 1BVN | -15.06 | -68.04 | 4.84 | -102.94 | 11.67 |
| 1EMV | -18.58 | -70.58 | 5.75 | -129.75 | 13.22 |
| 1FLE | -12.28 | -76.39 | 4.47 | -106.3 | 10.48 |
| 1GLA | -6.76 | -25.00 | 4.79 | -46.95 | 10.83 |
| 1KAC | -10.68 | -39.2 | 5.73 | -84.15 | 13.01 |
| 1R0R | -14.17 | -66.91 | 4.72 | -104.02 | 11.13 |
| 1YVB | -11.17 | -77.69 | 6.84 | -100.53 | 10.75 |
| 2HRK | -10.98 | -43.35 | 4.02 | -94.16 | 11.68 |
| 2OOB | -5.66 | -27.07 | 4.1 | -57.43 | 8.64 |
| 2OUL | -11.96 | -75.74 | 4.1 | -101.47 | 10.97 |
| 2SIC | -13.84 | -90.06 | 4.59 | -118.46 | 9.5 |
| 2SNI | -15.96 | -79.93 | 6.17 | -118.56 | 13.11 |
| 2UUY | -11.26 | -62.17 | 6.1 | -90.73 | 10.39 |
| 3BZD | -9.57 | -24.82 | 3.91 | -75.18 | 12 |
| 3SGB | -14.51 | -65.35 | 3.94 | -94.37 | 9.92 |

**Table S3.** MM-GBSA results obtained from the 4^th^ ns of MD Simulation for starting structures originated from MnM-Scheme-1.

| **PDB_ID** | **Experimental_dG** | **No_interfacial_water** | | **30_interfacial_waters** | |
| --- | --- | --- | --- | --- | --- |
|  |  | **∆G_bind_** | **Std. Dv.** | **∆G_bind_** | **Std. Dv.** |
| 1ACB | -13.1 | -60.9 | 4.2 | -108.4 | 12.3 |
| 1AVX | -12.5 | -69.3 | 5.7 | -85.9 | 11.2 |
| 1AY7 | -13.2 | -34.1 | 4.3 | -77.3 | 11.9 |
| 1BVN | -15.1 | -43.2 | 4.5 | -74.2 | 11.0 |
| 1EMV | -18.6 | -80.4 | 5.0 | -117.3 | 10.8 |
| 1FLE | -12.3 | -51.5 | 10.5 | -67.2 | 13.0 |
| 1GLA | -6.8 | -22.7 | 4.1 | -54.3 | 12.2 |
| 1KAC | -10.7 | -28.0 | 4.2 | -70.2 | 12.8 |
| 1R0R | -14.2 | -68.9 | 4.5 | -102.8 | 9.0 |
| 1YVB | -11.2 | -61.3 | 4.3 | -77.4 | 9.0 |
| 1ZHI | -9.1 | -31.5 | 5.1 | -59.7 | 9.4 |
| 2HLE | -10.1 | -59.6 | 7.0 | -83.4 | 14.2 |
| 2HRK | -11.0 | -45.6 | 4.5 | -83.2 | 12.1 |
| 2OOB | -5.7 | -25.1 | 3.4 | -46.0 | 9.2 |
| 2OUL | -12.0 | -73.6 | 5.5 | -98.2 | 11.5 |
| 2SIC | -13.8 | -83.5 | 5.4 | -109.8 | 10.4 |
| 2SNI | -16.0 | -77.4 | 4.2 | -118.9 | 11.3 |
| 2UUY | -11.3 | -44.5 | 4.5 | -85.5 | 11.3 |
| 3BZD | -9.6 | -35.8 | 4.2 | -92.8 | 12.0 |
| 3SGB | -14.5 | -53.6 | 4.0 | -86.8 | 9.7 |

**Table S4.** MM-GBSA results obtained from the 40 to 50 ns of MD Simulation for starting structures originated from MnM-Scheme-1.

| **PDB_ID** | **Experimental_dG** | **No_interfacial_water** | | **30_interfacial_waters** | |
| --- | --- | --- | --- | --- | --- |
|  |  | **∆G_bind_** | **Std. Dv.** | **∆G_bind_** | **Std. Dv.** |
| 1ACB | -13.1 | -56.2 | 4.8 | -105.2 | 12.2 |
| 1AVX | -12.5 | -67.5 | 6.1 | -86.7 | 10.3 |
| 1AY7 | -13.2 | -49.7 | 5.6 | -94.1 | 12.0 |
| 1BVN | -15.1 | -74.2 | 5.4 | -104.4 | 13.5 |
| 1EMV | -18.6 | -86.3 | 6.2 | -139.3 | 12.8 |
| 1FLE | -12.3 | -75.2 | 5.7 | -98.6 | 9.9 |
| 1GLA | -6.8 | -25.9 | 10.4 | -67.6 | 19.8 |
| 1KAC | -10.7 | -34.5 | 8.3 | -84.2 | 17.7 |
| 1R0R | -14.2 | -75.5 | 5.6 | -108.4 | 13.2 |
| 1YVB | -11.2 | -61.4 | 5.0 | -81.9 | 10.9 |
| 1ZHI | -9.1 | -46.0 | 5.6 | -80.1 | 11.6 |
| 2HLE | -10.1 | -53.9 | 6.8 | -83.2 | 11.5 |
| 2HRK | -11.0 | -47.2 | 4.8 | -86.1 | 12.1 |
| 2OOB | -5.7 | -29.7 | 3.1 | -53.4 | 10.9 |
| 2OUL | -12.0 | -74.0 | 4.3 | -102.5 | 10.9 |
| 2SIC | -13.8 | -86.4 | 5.2 | -113.3 | 11.4 |
| 2SNI | -16.0 | -79.9 | 4.7 | -126.7 | 11.4 |
| 2UUY | -11.3 | -40.5 | 4.6 | -78.4 | 10.8 |
| 3BZD | -9.6 | -27.1 | 4.4 | -65.3 | 11.4 |
| 3SGB | -14.5 | -67.0 | 5.1 | -93.9 | 11.1 |

**Table S5.** Pairwise per-residue energy decomposition of the MM-GBSA results. For the residue pairs contributing more than -3 kcal/mol difference (between MnM-Scheme-1 and Scheme-0, green bars in Figure-5) and more than +3 kcal/mol (between MnM-Scheme-1 and Scheme-0, red bars in Figure-5) are listed. For 2OOB and 2OUL no residue pairs contributed more than ±3 kcal/mol difference between the schemes (denoted as N/A).

| **PPI** | **Resid1** | **Resid2** | **Difference**  **(MnM-Scheme-1 – Scheme-0)** |
| --- | --- | --- | --- |
| 1ACB | SER195 | LEU281 | -3.1 |
| 1ACB | PHE41 | LEU283 | -3.1 |
| 1ACB | PHE39 | TYR285 | -3.1 |
| 1ACB | LEU281 | HIS57 | 3.6 |
| 1AVX | TYR131 | ASN236 | -3.3 |
| 1AVX | ASP171 | ARG286 | 4.0 |
| 1AVX | SER129 | GLU235 | 4.1 |
| 1AVX | ARG288 | HIE23 | 5.2 |
| 1GLA | ARG480 | GLU551 | -5.0 |
| 1GLA | ARG477 | ASP517 | -3.7 |
| 1GLA | GLU551 | GLN335 | 4.1 |
| 1GLA | TYR479 | GLU576 | 5.0 |
| 1GLA | THR474 | ASP517 | 6.2 |
| 1GLA | THR475 | ASP517 | 9.5 |
| 1ZHI | GLU53 | ARG238 | -5.6 |
| 1ZHI | THR57 | GLU228 | -4.5 |
| 1ZHI | ASN109 | GLU210 | -3.8 |
| 1ZHI | ALA55 | ARG215 | -3.6 |
| 2HLE | LYS141 | GLU286 | -6.6 |
| 2HLE | ARG48 | LYS270 | -5.7 |
| 2HLE | ASP21 | VAL269 | -3.7 |
| 2HLE | SER38 | PHE271 | 3.1 |
| 2HLE | LYS270 | THR19 | 3.5 |
| 2HLE | SER38 | THR272 | 3.5 |
| 2HLE | GLU35 | LYS218 | 4.0 |
| 2HLE | ASP21 | LYS270 | 5.1 |
| 2HLE | GLU35 | LYS274 | 5.9 |
| 2HLE | GLU51 | SER279 | 6.0 |
| 1BVN | SER310 | ASP517 | -8.1 |
| 1BVN | ASP517 | SER310 | -8.0 |
| 1BVN | HIS305 | TYR508 | 3.8 |
| 1BVN | TYR508 | HIS305 | 3.8 |
| 1AY7 | ASP131 | ARG40 | -4.1 |
| 1EMV | LYS97 | GLU170 | -11.1 |
| 1EMV | ARG96 | GLU160 | -6.8 |
| 1EMV | LYS89 | GLU170 | -4.1 |
| 1FLE | ASP48 | ARG261 | -6.2 |
| 1FLE | ARG211 | GLU276 | 4.5 |
| 1FLE | ASP86 | ARG261 | 6.3 |
| 1KAC | LYS49 | ASP246 | 2.9 |
| 1R0R | SER220 | LEU287 | -5.0 |
| 1R0R | GLY101 | LYS282 | -3.9 |
| 1R0R | PHE188 | TYR289 | -3.6 |
| 1R0R | ASN217 | TYR289 | -3.4 |
| 1R0R | ASN154 | LEU287 | -3.3 |
| 1R0R | GLY126 | CYS285 | -3.2 |
| 1YVB | LEU172 | ARG242 | -4.7 |
| 1YVB | GLN171 | ARG242 | -4.4 |
| 1YVB | ASP154 | ARG288 | -3.1 |
| 1YVB | ASP234 | ARG242 | 4.1 |
| 2OOB | N/A | N/A | N/A |
| 2OUL | N/A | N/A | N/A |
| 2SIC | GLN103 | ASP337 | 4.2 |
| 2SNI | GLU156 | ARG337 | -3.1 |
| 2SNI | GLU316 | HIS64 | 9.1 |
| 2UUY | THR243 | TYR22 | 3.3 |
| 2UUY | GLY196 | LYS239 | 4.3 |
| 2UUY | ASP171 | LYS239 | 7.5 |

**Table S6.** MM-GBSA results obtained from the 4^th^ ns of MD Simulation for starting structures originated from MnM-Scheme-2.

| **PDB_ID** | **Experimental_dG** | **No_interfacial_water** | | **30_interfacial_waters** | |
| --- | --- | --- | --- | --- | --- |
|  |  | **∆G_bind_** | **Std. Dv.** | **∆G_bind_** | **Std. Dv.** |
| 1ACB | -13.1 | -44.8 | 3.5 | -92.6 | 11.4 |
| 1AVX | -12.5 | -63.8 | 8.6 | -94.0 | 12.7 |
| 1AY7 | -13.2 | -33.4 | 4.1 | -68.5 | 12.2 |
| 1BVN | -15.1 | -36.2 | 7.6 | -57.9 | 16.0 |
| 1EMV | -18.6 | -47.4 | 4.8 | -89.9 | 14.6 |
| 1FLE | -12.3 | -73.8 | 4.1 | -104.4 | 10.4 |
| 1GLA | -6.8 | -115.5 | 10.8 | -151.8 | 15.8 |
| 1KAC | -10.7 | -35.2 | 5.3 | -75.0 | 12.6 |
| 1R0R | -14.2 | -60.7 | 5.6 | -94.3 | 12.6 |
| 1YVB | -11.2 | -69.7 | 6.6 | -91.2 | 12.5 |
| 1ZHI | -9.1 | -23.4 | 4.0 | -55.0 | 10.7 |
| 2HLE | -10.1 | -24.3 | 5.1 | -43.2 | 9.9 |
| 2HRK | -11.0 | -41.9 | 4.4 | -80.9 | 11.7 |
| 2OOB | -5.7 | -26.6 | 3.3 | -51.2 | 7.5 |
| 2OUL | -12.0 | -59.9 | 3.8 | -88.0 | 11.8 |
| 2SIC | -13.8 | -54.5 | 3.8 | -78.0 | 10.4 |
| 2SNI | -16.0 | -79.4 | 4.9 | -125.0 | 12.1 |
| 2UUY | -11.3 | -29.9 | 3.5 | -70.9 | 10.8 |
| 3BZD | -9.6 | -31.2 | 4.4 | -65.2 | 11.0 |
| 3SGB | -14.5 | -41.6 | 4.1 | -72.8 | 9.6 |

**Table S7.** MM-GBSA results obtained from the 40 to 50ns of MD Simulation for starting structures originated from MnM-Scheme-2.

| **PDB_ID** | **Experimental_dG** | **No_interfacial_water** | | **30_interfacial_waters** | |
| --- | --- | --- | --- | --- | --- |
|  |  | **∆G_bind_** | **Std. Dv.** | **∆G_bind_** | **Std. Dv.** |
| 1ACB | -13.1 | -45.8 | 4.4 | -88.8 | 10.4 |
| 1AVX | -12.5 | -89.6 | 8.9 | -125.2 | 16.0 |
| 1AY7 | -13.2 | -17.0 | 4.5 | -42.0 | 11.3 |
| 1BVN | -15.1 | -39.6 | 6.2 | -69.0 | 14.4 |
| 1EMV | -18.6 | -52.2 | 6.2 | -81.4 | 9.8 |
| 1FLE | -12.3 | -74.5 | 5.0 | -98.0 | 11.0 |
| 1GLA | -6.8 | -152.5 | 8.3 | -186.6 | 12.5 |
| 1KAC | -10.7 | -34.2 | 6.5 | -82.7 | 14.5 |
| 1R0R | -14.2 | -64.4 | 6.2 | -98.3 | 9.6 |
| 1YVB | -11.2 | -61.5 | 7.6 | -81.8 | 12.6 |
| 1ZHI | -9.1 | -47.2 | 4.9 | -153.6 | 14.0 |
| 2HLE | -10.1 | -55.4 | 7.3 | -74.9 | 10.6 |
| 2HRK | -11.0 | -41.7 | 4.3 | -85.5 | 12.4 |
| 2OOB | -5.7 | -28.9 | 3.7 | -55.4 | 9.6 |
| 2OUL | -12.0 | -76.3 | 4.9 | -96.2 | 11.9 |
| 2SIC | -13.8 | -57.5 | 4.2 | -83.8 | 11.2 |
| 2SNI | -16.0 | -80.5 | 5.6 | -133.7 | 16.9 |
| 2UUY | -11.3 | -40.3 | 5.6 | -75.9 | 11.1 |
| 3BZD | -9.6 | -27.8 | 4.9 | -68.2 | 12.6 |
| 3SGB | -14.5 | -50.1 | 4.8 | -82.8 | 9.3 |

**Supplementary Figures**


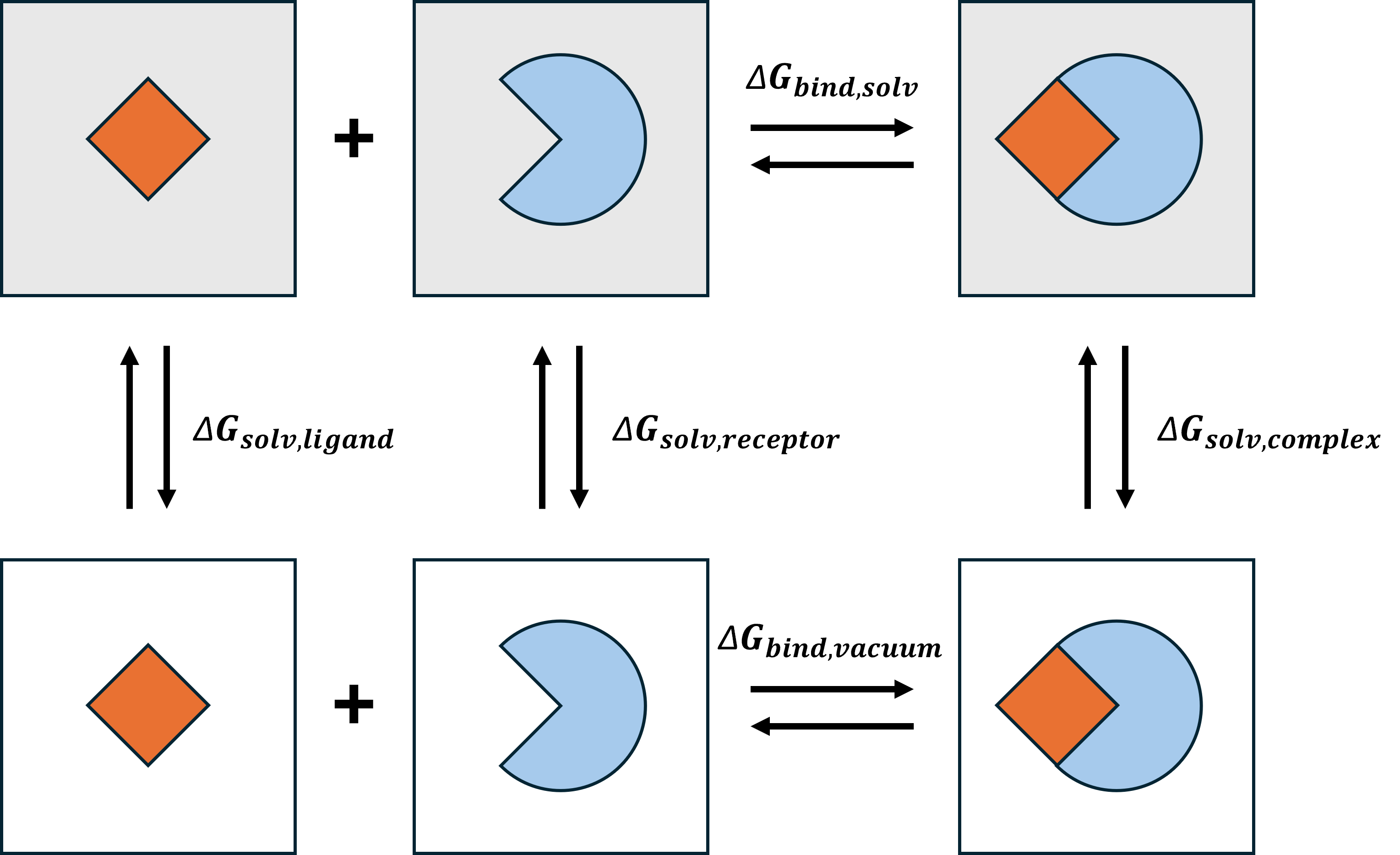


**Figure S1.** Schematic representation of the thermodynamic cycle for the free energy calculation using MM-GBSA method.

**

**

**Figure S2.** C-alpha RMSD for the PPI complexes (stating with crystal structures) calculated for the 4^th^ ns. The timescale is in picoseconds (ps) in X-axis and RMSD is in angstroms in Y-axis for all the PPIs.

**
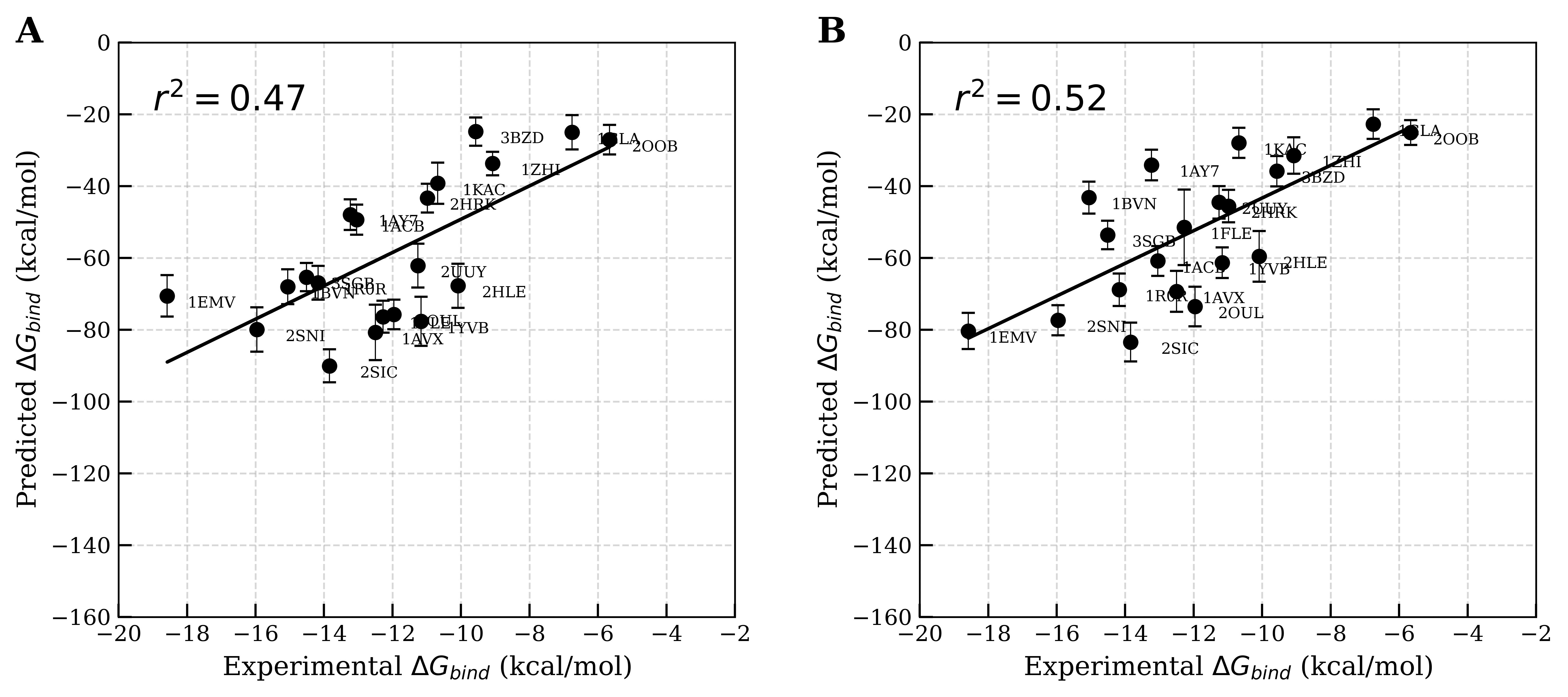
**

**Figure S3.** Correlation between experimental Δ*G_bind_* and predicted binding free energies calculated using MM-GBSA. The binding free energies were calculated from 100 frames extracted at the 4^th^ ns of the MD trajectories, where the starting structures originated from the X-ray crystal structures obtained from Scheme-0 (A), and MnM-Scheme-1 approach (B).


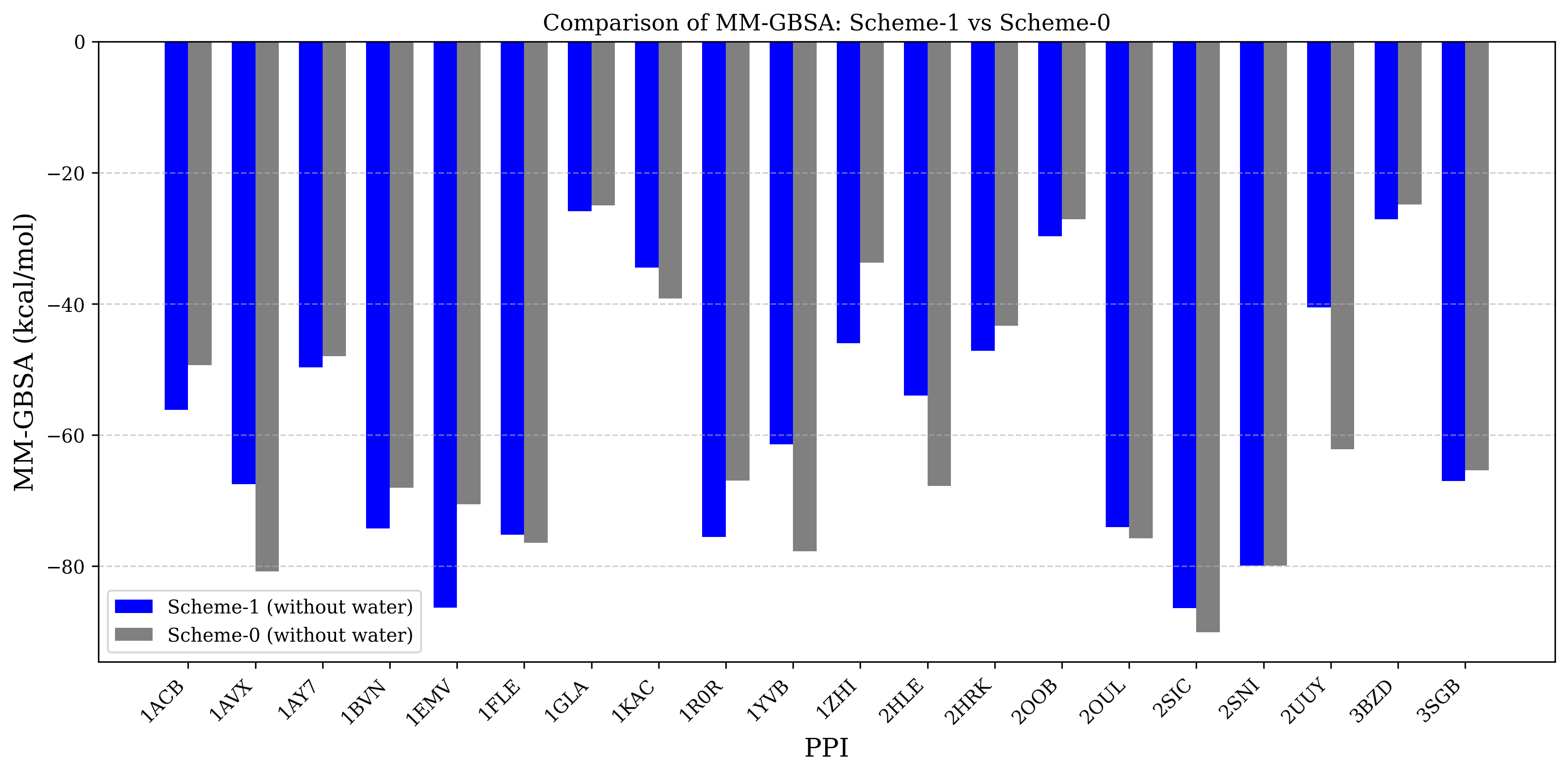


**Figure S4:** MM-GBSA without interfacial waters obtained from Scheme-0 vs Scheme-1.


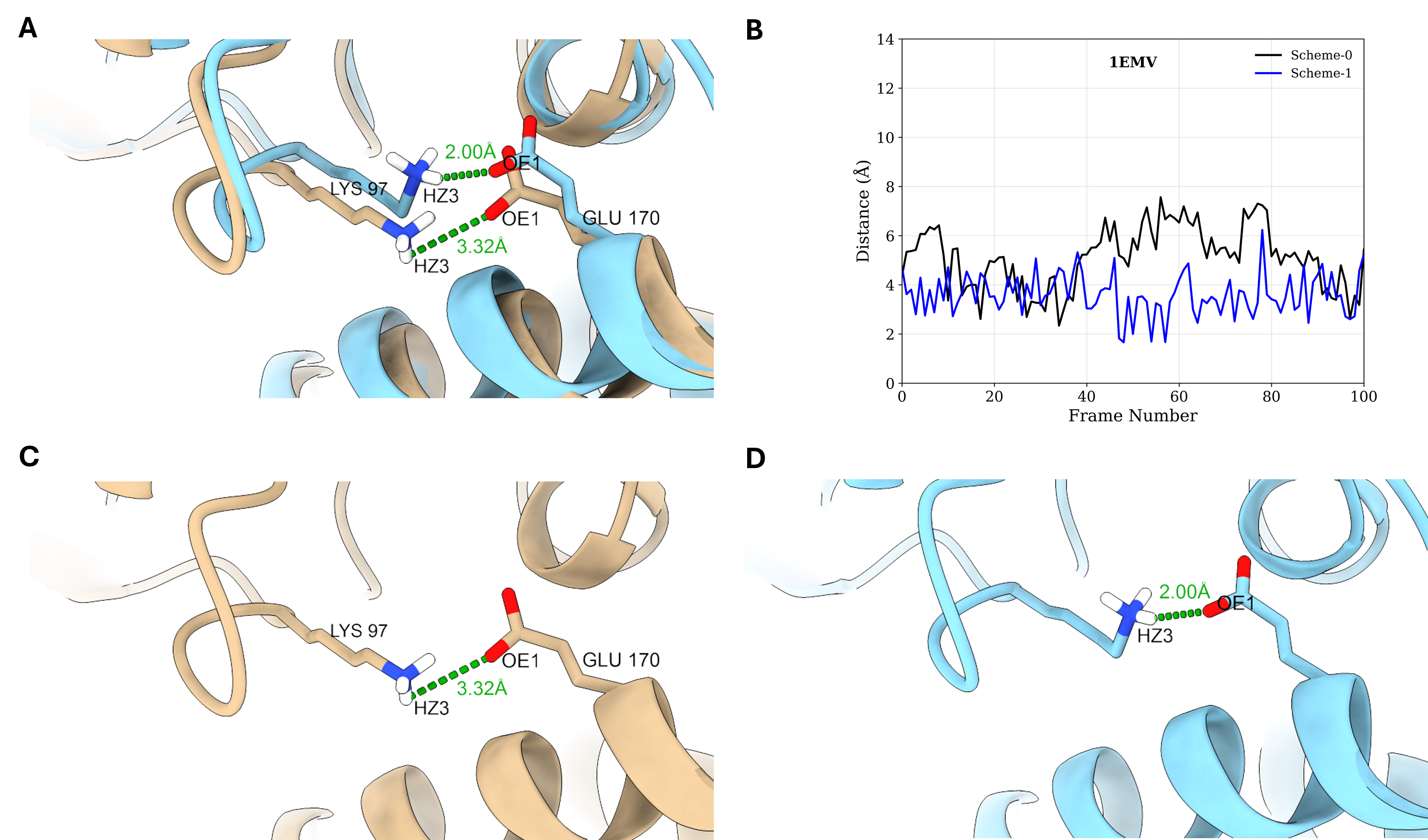


Figure S5. A. Overlay of the representative structures of 1EMV from Scheme-0 (beige) vs MnM-Scheme-1 (cyan) 40 to 50 ns. B. Distance between atom pairs of GLU53 OE2 and ARG238 HH11 in the 100 extracted frames for MMGBSA calculation. C and D. Distance between atom pairs of GLU53 OE2 and ARG238 HH11 in the representative structure of Scheme-0 and MnM-Scheme-1 respectively.


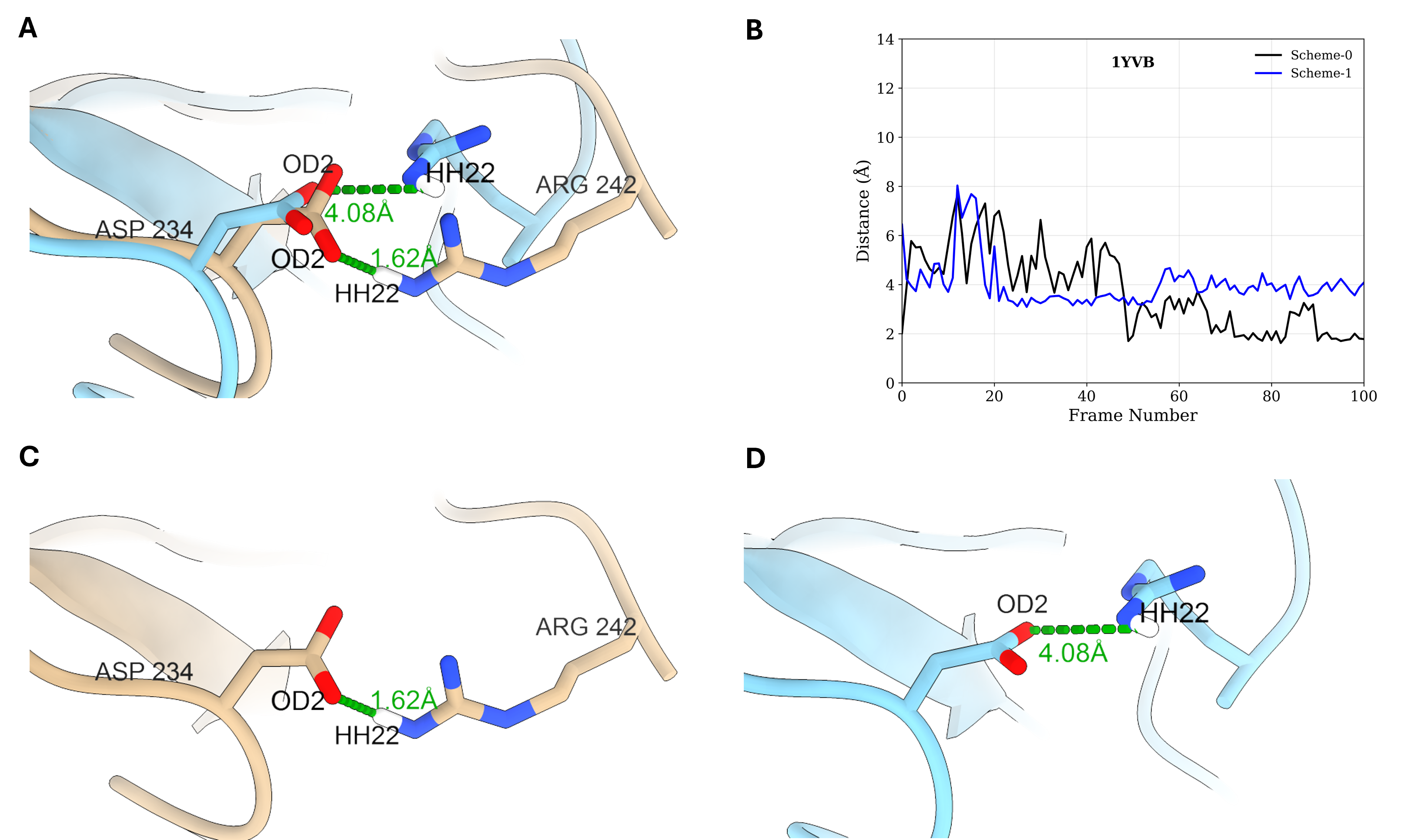


Figure S6. A. Overlay of the representative structures of 1YVB from Scheme-0 (beige) vs MnM-Scheme-1 (cyan) 40 to 50 ns. B. Distance between atom pairs of ASP234 OD2 and ARG242 HH22 in the 100 extracted frames for MMGBSA calculation. C and D. Distance between atom pairs of ASP234 OD2 and ARG242 HH22 in the representative structure of Scheme-0 and MnM-Scheme-1 respectively.


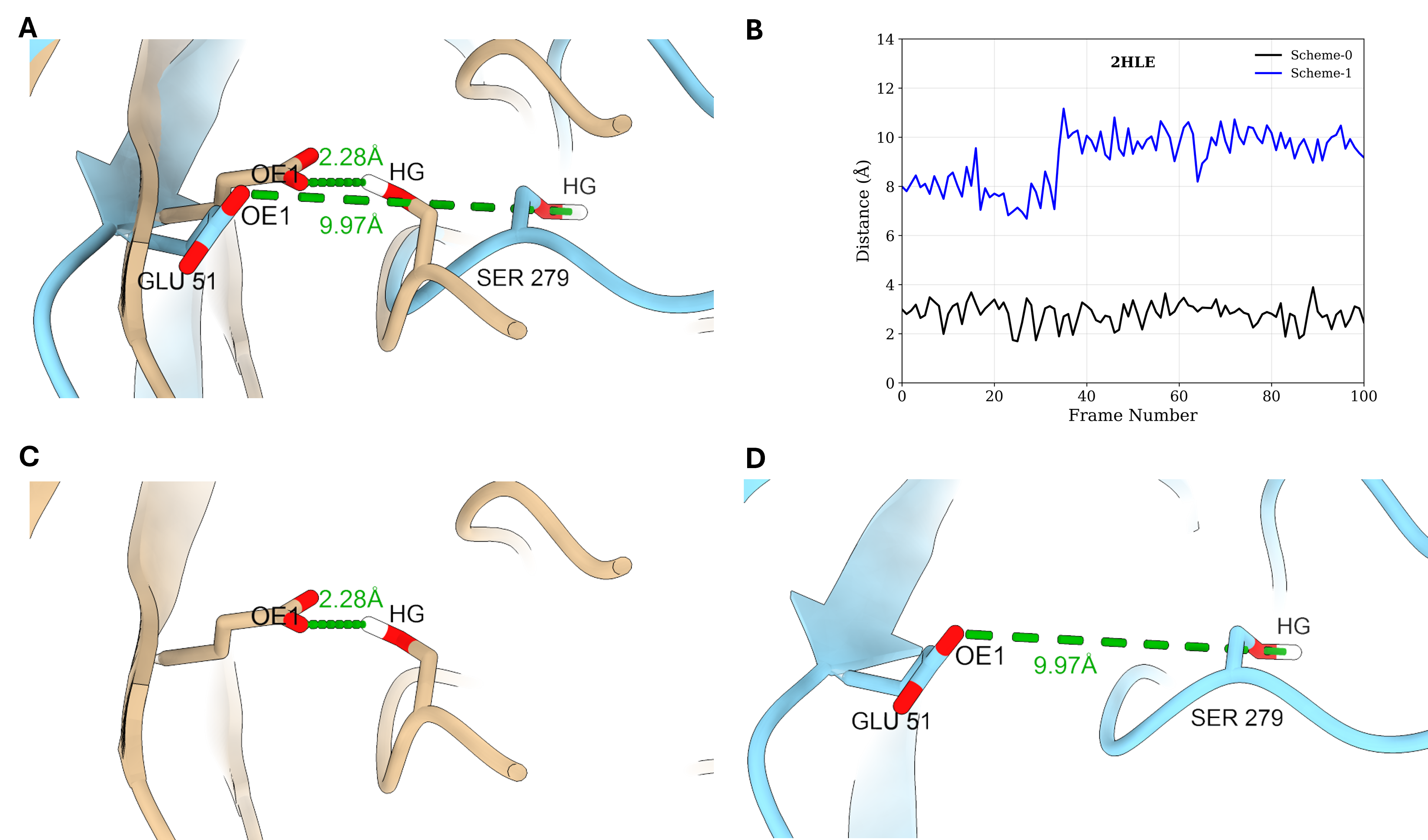


Figure S7. A. Overlay of the representative structures of 2HLE from Scheme-0 (beige) vs MnM-Scheme-1 (cyan) 40 to 50 ns. B. Distance between atom pairs of GLU51 OE1 and SER279 HG in the 100 extracted frames for MMGBSA calculation. C and D. Distance between atom pairs of GLU51 OE1 and SER279 HG in the representative structure of Scheme-0 and MnM-Scheme-1 respectively.


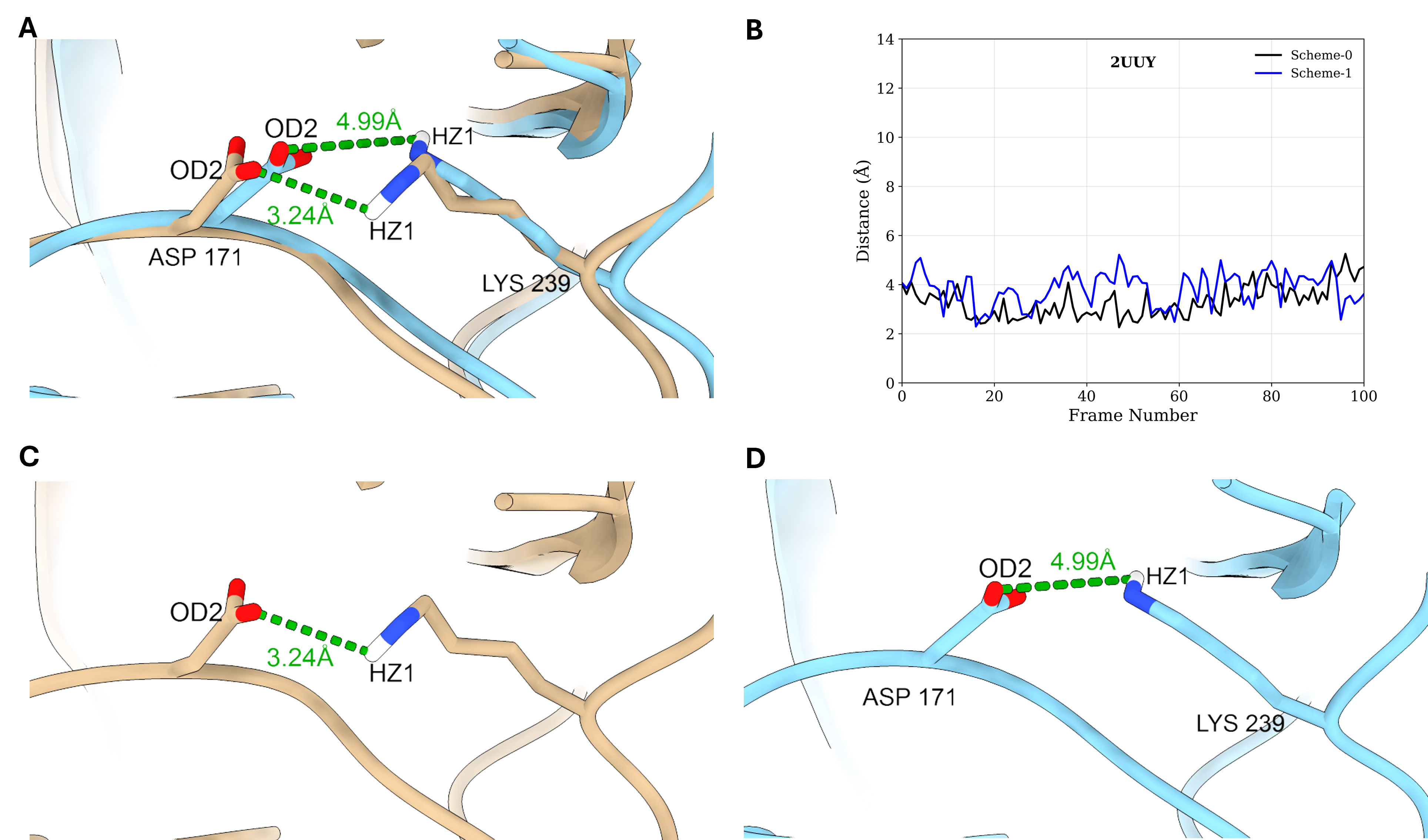


Figure S8. A. Overlay of the representative structures of 2UUY from Scheme-0 (beige) vs MnM-Scheme-1 (cyan) 40 to 50 ns. B. Distance between atom pairs of ASP171 OD2 and LYS239 HZ1 in the 100 extracted frames for MMGBSA calculation. C and D. Distance between atom pairs of ASP171 OD2 and LYS239 HZ1 in the representative structure of Scheme-0 and MnM-Scheme-1 respectively.

**
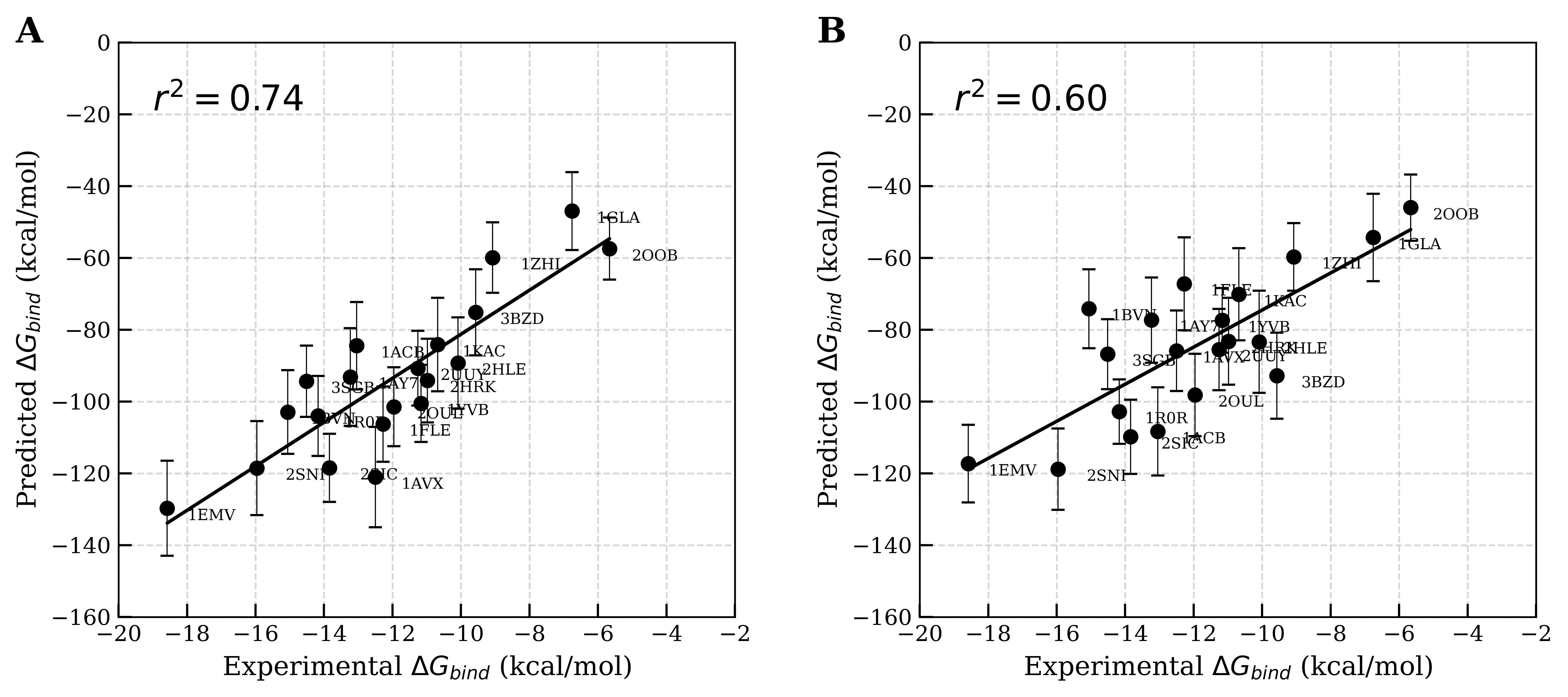
**

**Figure S9.** Correlation between experimental Δ*G_bind_* and predicted binding free energies calculated using MM-GBSA including 30 interfacial waters. The binding free energies were calculated from 100 frames extracted at the 4^th^ ns of the MD trajectories, where the starting structures originated from the X-ray crystal structures obtained from Scheme-0 (A), and MnM-Scheme-1 approach (B).

**
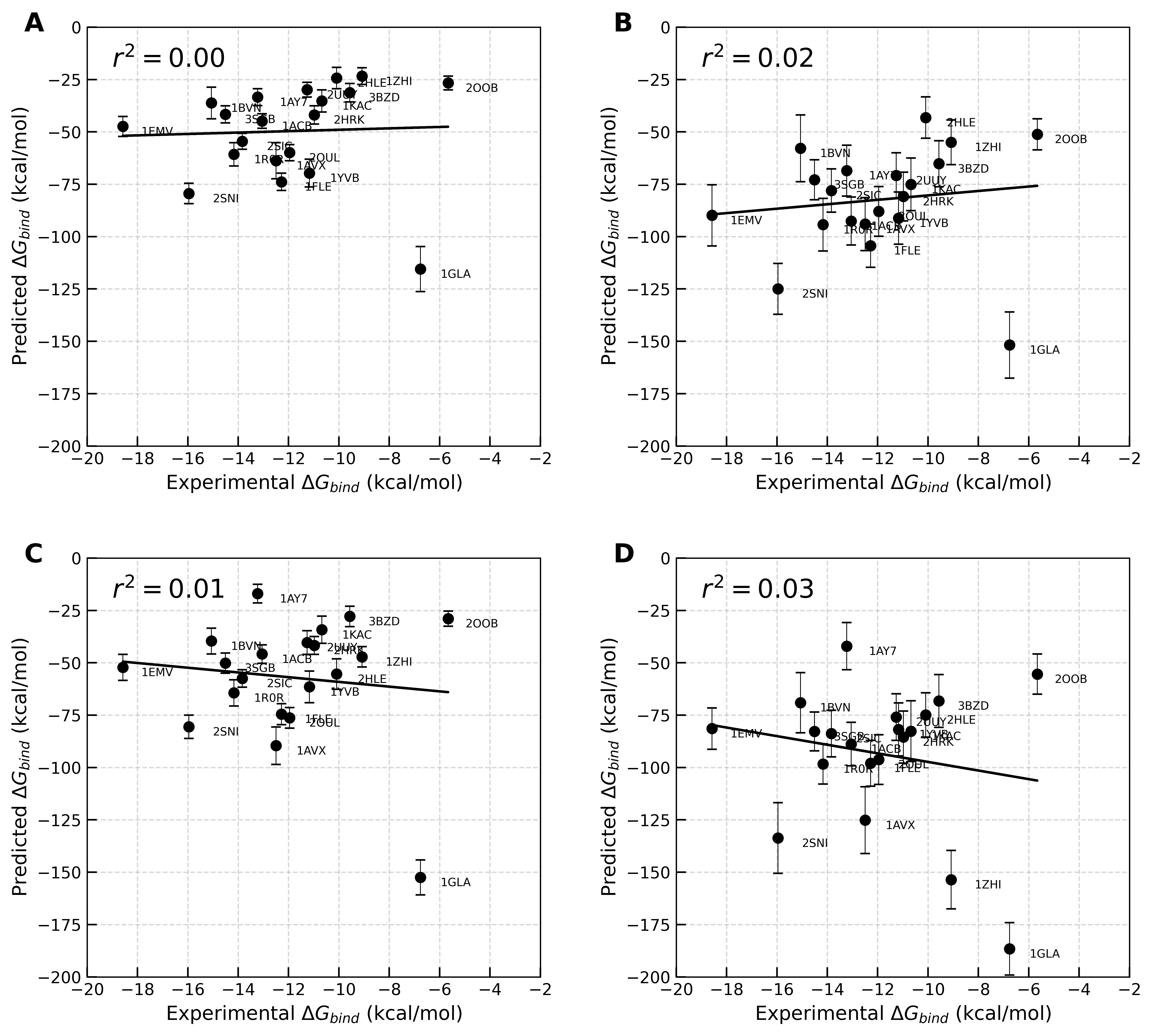
**

**Figure S10.** Correlation between experimental Δ*G_bind_* and predicted binding energies obtained from MnM scheme 2 MM-GBSA analysis for the 4^th^ ns timeframe without including interfacial waters (A) and with 30 interfacial waters (B), 40 to 50ns timeframe without including interfacial waters (C) and with 30 interfacial waters (D).

**
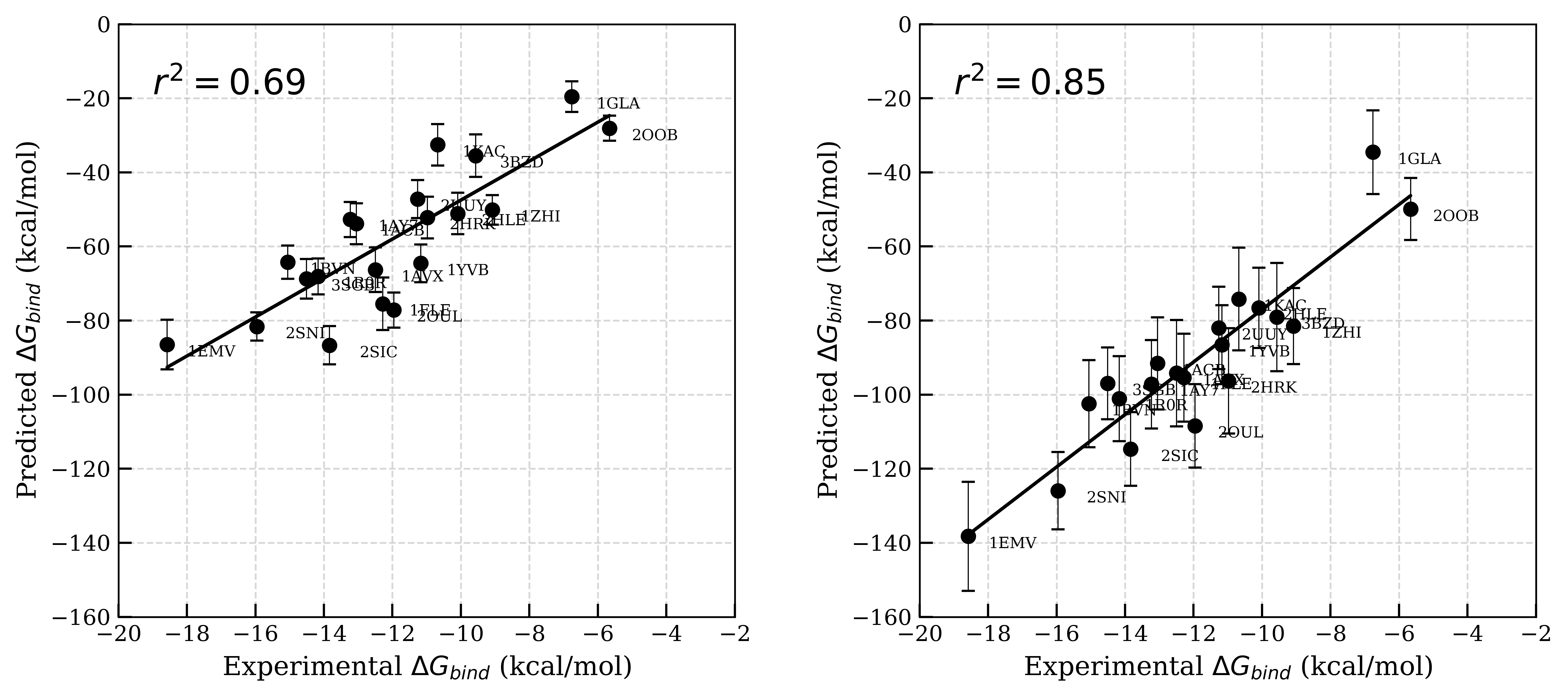
**

**Figure S11.** Correlation between experimental Δ*G_bind_* and predicted binding free energies calculated using MM-GBSA for the second run. The binding free energies were calculated for 100 frames including 30 interfacial waters extracted from the MD trajectories at the 4^th^ ns starting from the crystal structures (Scheme-0) (A) and 40 to 50ns starting from structures originated through the MnM-Scheme-1 approach (B).


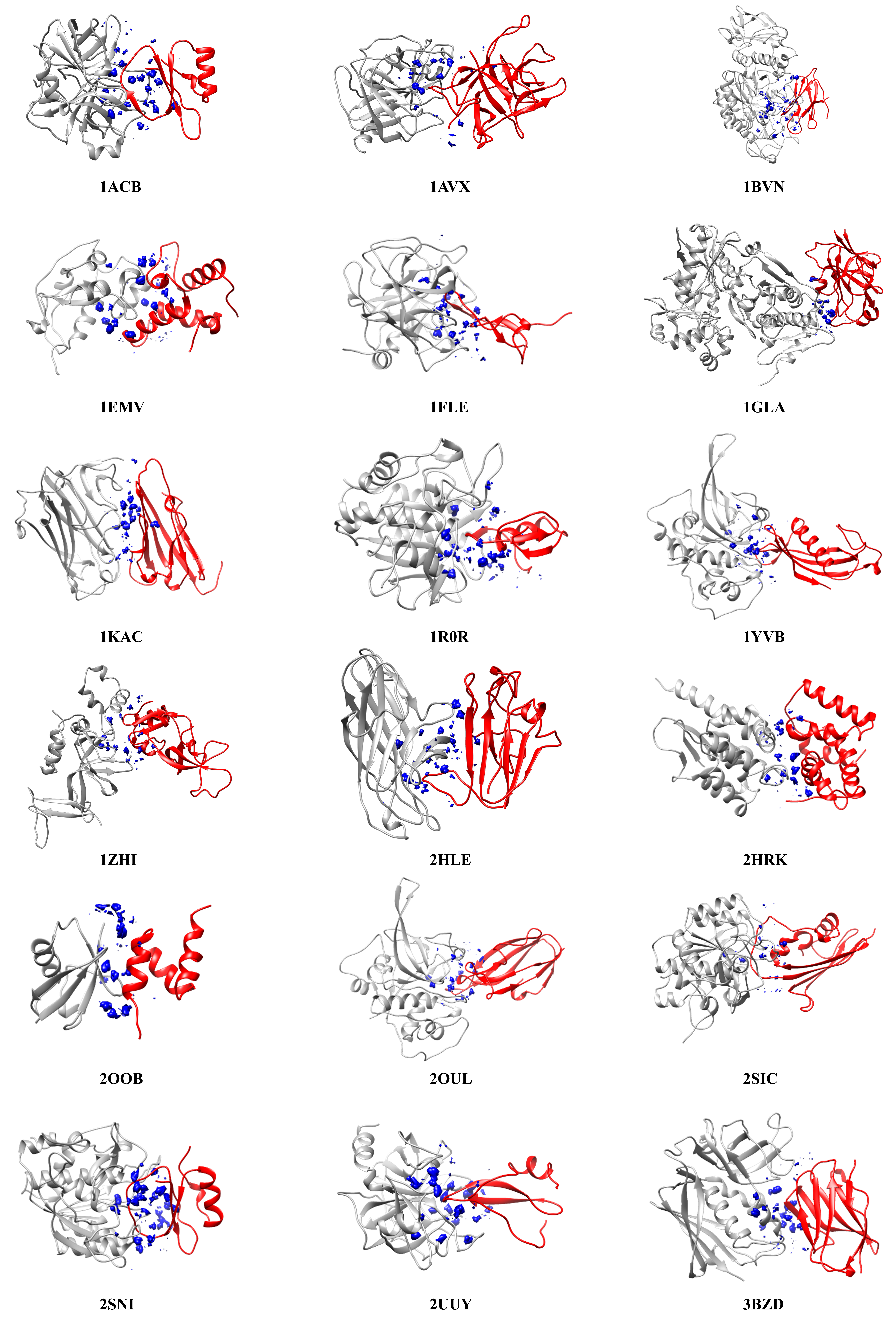


**Figure S12.** Water density plots obtained by grid analysis of the MnM-Scheme-1 complexes for 40 to 50ns of MD trajectory visualized with Chimera (step 1, level 8 for H and level 5 for O).





**Figure S13.** Side chain RMSF comparison between the starting structures. 4^th^ ns simulation for crystal structures (black line) and interfacial residues is highlighted in black dots. RMSF for Scheme-1 interfacial residues (40-50ns) is highlighted as green diamonds and Scheme-2 interfacial residues (40-50ns) is highlighted as red triangles. X-axis represents the residue numbers and RMSF is shown in Y-axis.
